# Long Noncoding RNA PCALRx Interacts with Pyruvate Carboxylase to Drive Multi‐Organ Developmental Toxicity in Zebrafish Embryos Exposed to Amoxicillin

**DOI:** 10.1002/advs.76937

**Published:** 2026-08-05

**Authors:** Yixue Yao, Ping Jiang, Xinli Zhou, Mengying Yin, Qiong Luo, Hui Wang

**Affiliations:** ^1^ Department of Pharmacology Wuhan University Taikang School of Medicine (School of Basic Medical Sciences) Wuhan China; ^2^ Hubei Provincial Key Laboratory of Developmentally Originated Disease Wuhan China

**Keywords:** amoxicillin, antibiotic pollution, developmental toxicity, long noncoding RNA, pyruvate carboxylase

## Abstract

Amoxicillin is widely used in human and veterinary medicine and is increasingly detected in aquatic systems, raising concerns regarding its potential adverse effects on sensitive developmental stages. Here, a zebrafish embryonic amoxicillin exposure (EAE) model was used to quantify dose‐ and time‐dependent developmental outcomes and to elucidate underlying metabolic–epigenetic mechanisms. EAE induced dose‐ and time‐dependent increases in mortality and malformation rates, and significantly impaired differentiation of liver, interrenal gland, heart and brain, accompanied by mitochondrial structural damage and reduction in ATP levels. Multi‐omics integration identified a previously uncharacterized long noncoding RNA, PCALRx, which was associated with pyruvate carboxylase (PC) and negatively regulated PC protein stability. PCALRx promoted ubiquitination‐mediated degradation of PC, resulting in impaired mitochondrial metabolic function and subsequent multi‐organ differentiation defects. Knockdown of PCALRx mitigated mitochondrial dysfunction and improved EAE‐induced developmental defects. Finally, a drug‐repurposing strategy nominated thiamine (vitamin B1) as a metabolic modulator capable of mitigating EAE‐induced developmental changes in zebrafish larvae. This protective effect was further validated in a mouse model of prenatal amoxicillin exposure. Together, these findings reveal an lncRNA‐mediated regulatory mechanism involving PC ubiquitination and mitochondrial metabolic disruption in amoxicillin‐associated developmental toxicity and provide a potential intervention strategy relevant to environmental amoxicillin exposure.

## Graphical abstract

Embryonic amoxicillin exposure disrupts multi‐organ development in zebrafish larvae through a lncRNA–metabolic enzyme regulatory axis. PCALRx associates with pyruvate carboxylase, promotes PC protein ubiquitination, and impairs mitochondrial energy metabolism, while vitamin B1 partially restores PC‐centered metabolic function and developmental outcomes.

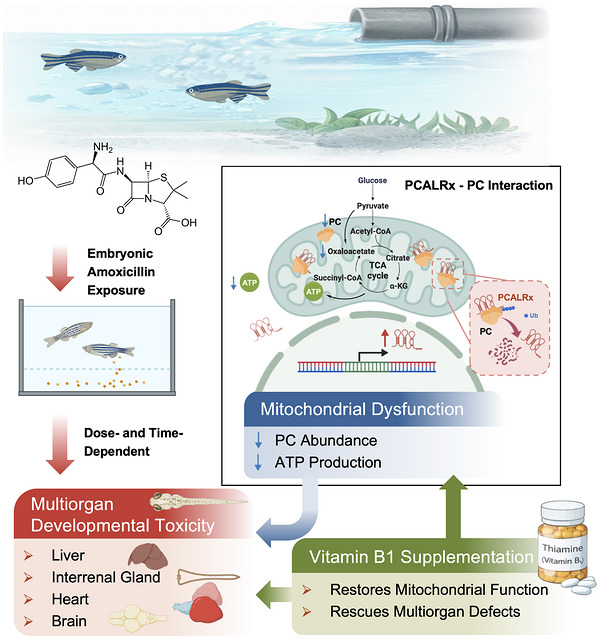

## 1 Introduction

The widespread use of antibiotics in both clinical and livestock settings has led to their continuous presence in the environment [1, 2, 3]. These compounds can re‐enter the human body through food chains and drinking water, making them a significant concern in environmental science and public health [4]. Numerous antibiotics are frequently detected in surface waters, groundwater, and animal‐derived foods [5, 6], and are considered “emerging environmental pollutants” with potential long‐term effects on ecosystems and human health through low‐dose exposure [7]. Amoxicillin, a commonly used β‐lactam antibiotic for both humans and animals [8], is widely detected in various aquatic environments, raising concerns about its ecological risks [9, 10, 11].

Pregnant women are more susceptible to infections and face increased risks of adverse pregnancy outcomes [12, 13]. Clinically, amoxicillin is commonly used during pregnancy due to its rapid absorption [14], high bioavailability [15], and good distribution in various organs, including the liver, lungs, prostate, and muscles [16], making it a preferred treatment for various infectious diseases during pregnancy [17, 18, 19, 20]. However, studies have confirmed that amoxicillin can cross the placenta, entering the fetal circulation and amniotic fluid [21, 22]. Epidemiological data also suggest that prenatal exposure to amoxicillin may be associated with an increased risk of fetal malformations [23, 24, 25]. Currently, there is a lack of systematic clarification regarding whether amoxicillin induces multi‐organ developmental toxicity and its potential shared mechanisms.

Embryonic development is highly dependent on energy supply, with mitochondrial metabolic homeostasis—particularly the efficiency of the tricarboxylic acid cycle (TCA) and ATP synthesis—playing a central role in organ differentiation and functional maturation. Disruptions to energy metabolism during this critical stage may become key targets for developmental toxicity induced by external chemicals and pharmaceuticals [26, 27]. Recent studies suggest that prenatal or embryonic exposure to antibiotics may disrupt energy metabolism, endocrine balance, and organ differentiation processes, potentially affecting the developmental trajectory and long‐term health of offspring [28]. Although previous studies have reported organ‐specific developmental toxicity of amoxicillin, including effects on the nervous system, lung alveolar development, and liver function [29, 30, 31], most of these findings are limited to single‐organ or phenotype‐focused investigations. A systematic understanding of whether a shared mechanism underlies multi‐organ developmental impairment remains lacking.

Furthermore, long non‐coding RNAs (lncRNAs), which bridge environmental exposure with metabolic regulation, play important roles in gene expression modulation at both the nuclear and cytoplasmic levels [32, 33]. They can interact with RNA‐binding proteins (RBPs) or metabolism‐related proteins and localize to mitochondria or other organelles, reshaping metabolic flux [34]. Some studies indicate that lncRNAs directly interact with metabolic proteins to regulate mitochondrial function, energy flux, and cellular differentiation states [35], playing key roles under developmental and stress conditions. Notably, certain lncRNAs have been identified as targeting TCA cycle‐related enzymes, affecting pyruvate metabolism and mitochondrial metabolic reprogramming [36, 37], though their role in antibiotic‐induced developmental toxicity remains poorly understood.

In recent years, the “drug repurposing” strategy, which leverages the advantages of approved drugs in terms of safety, pharmacokinetics, and clinical translation, has provided new pathways for the rapid identification of metabolic modulators with developmental protective effects. This approach has shown positive progress in the study of metabolic disorders, neurodevelopmental abnormalities, and mitochondrial dysfunction [38, 39], offering a safe and feasible route for intervention and risk mitigation in developmental toxicity.

In this study, we used the zebrafish embryonic amoxicillin exposure (EAE) model to systematically evaluate the effects of amoxicillin on multi‐organ development and differentiation. Through a series of high‐throughput sequencing and in vivo and in vitro experiments, we identified the key lncRNA PCALRx (Pyruvate Carboxylase‐Associated LncRNA x), and further explored its potential role in mediating the multi‐organ differentiation defects caused by EAE. Finally, we employed a drug repurposing strategy to screen for effective intervention drugs targeting shared toxicological mechanisms. This study confirms the multi‐organ developmental toxicity of amoxicillin, identifies common toxicological targets, and proposes effective intervention drugs, providing both theoretical and experimental support for the safe clinical use of drugs.

## 2 Results

### 2.1 EAE‐Induced Zebrafish Larvae Morphological Development Exhibits Dose‐ and Time‐Dependent Changes

**FIGURE 1:**
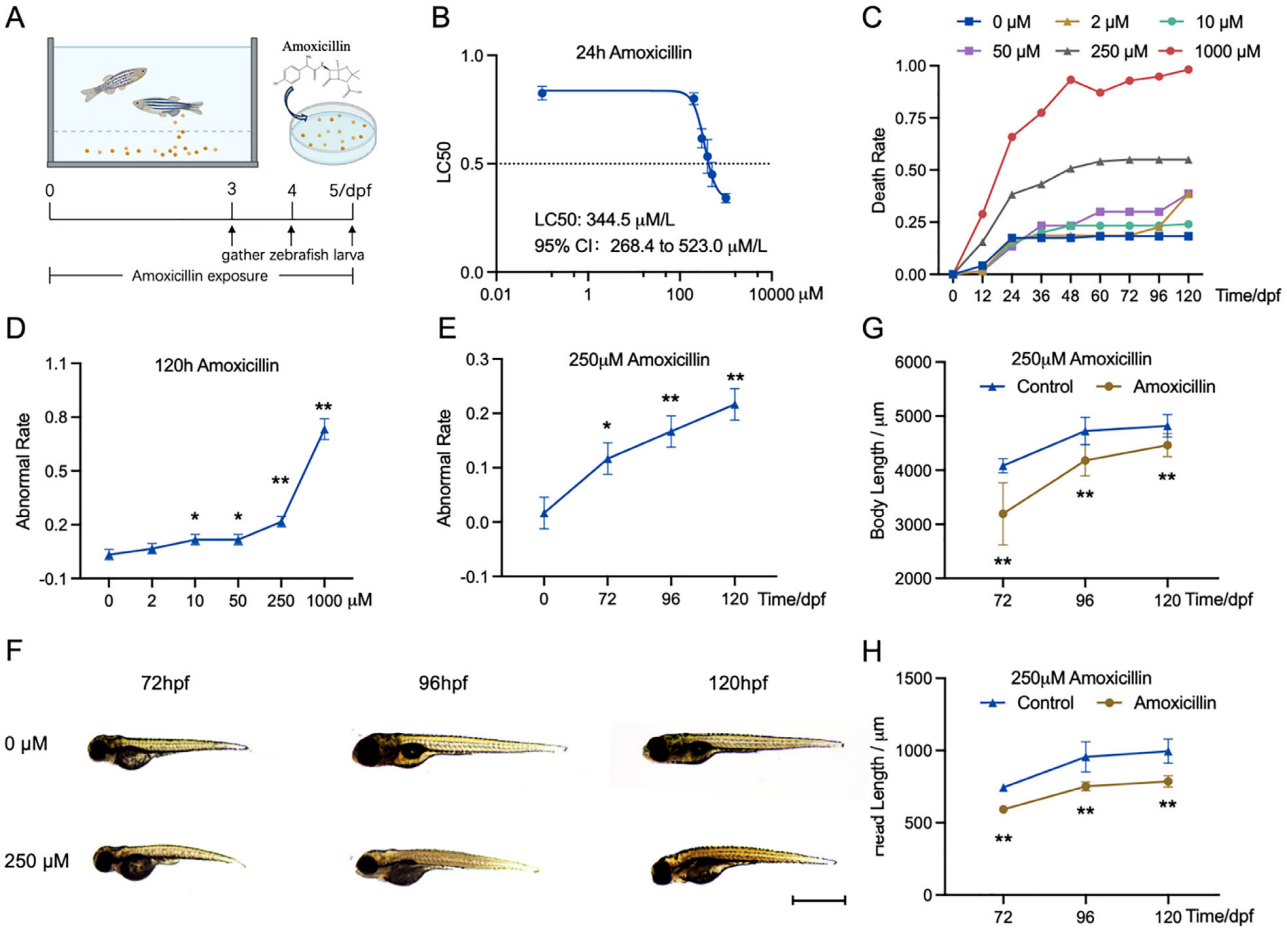
Effects of EAE on zebrafish larvae systemic development. (A) Experimental design for EAE. (B) 24 h LC_50_ of amoxicillin. (C) Cumulative death rate (0–120 hpf) across EAE concentrations. (D) Abnormal rate at 120 hpf across concentrations. (E) Abnormal rate of 250 µm EAE across exposure durations. (F) Representative bright‐field images of EAE zebrafish larvae. Scale bar, 1000 µm. (G) Body length and (H) head length at 120 hpf. n = 3 clutches (50 embryos each) for death rate; n = 6 for length. Data are mean ± S.E.M. ^*^ *P<*0.05, ^**^ *P*<0.01 *vs*. control, one‐way analysis of variance (ANOVA), followed by Tukey's multiple comparison test. Death rate and abnormal rate were analyzed using two‐way repeated measures ANOVA.

First, we observed the effects of EAE on zebrafish larvae overall morphological development at different concentrations (0, 2, 10, 50, 250, and 1000 µm) and exposure durations (0‐72, 0–96, and 0–120 hpf) (Figure 1A). The results showed that the 24‐h acute exposure lethal concentration 50% (LC_50_) was 344 µm (95% S.E.M. = 268–523 µm, Figure 1B). Death and abnormal rates of the larvae increased with increasing concentrations and exposure durations of EAE (*P*<0.05, *P*<0.01, Figure 1C–E, Figure S1A). Bright‐field imaging of the zebrafish larvae indicated that EAE led to concentration‐ and time‐dependent changes in overall morphology (Figure 1F; Figure S1B), with significant reductions in both body and head lengths (*P*<0.01, Figure 1G,H). These results suggest that EAE can cause overall developmental damage in zebrafish larvae, with clear dose‐ and time‐dependent effects.

### 2.2 EAE Induces Multi‐Organ Morphological and Functional Developmental Alterations in Zebrafish Larvae

**FIGURE 2:**
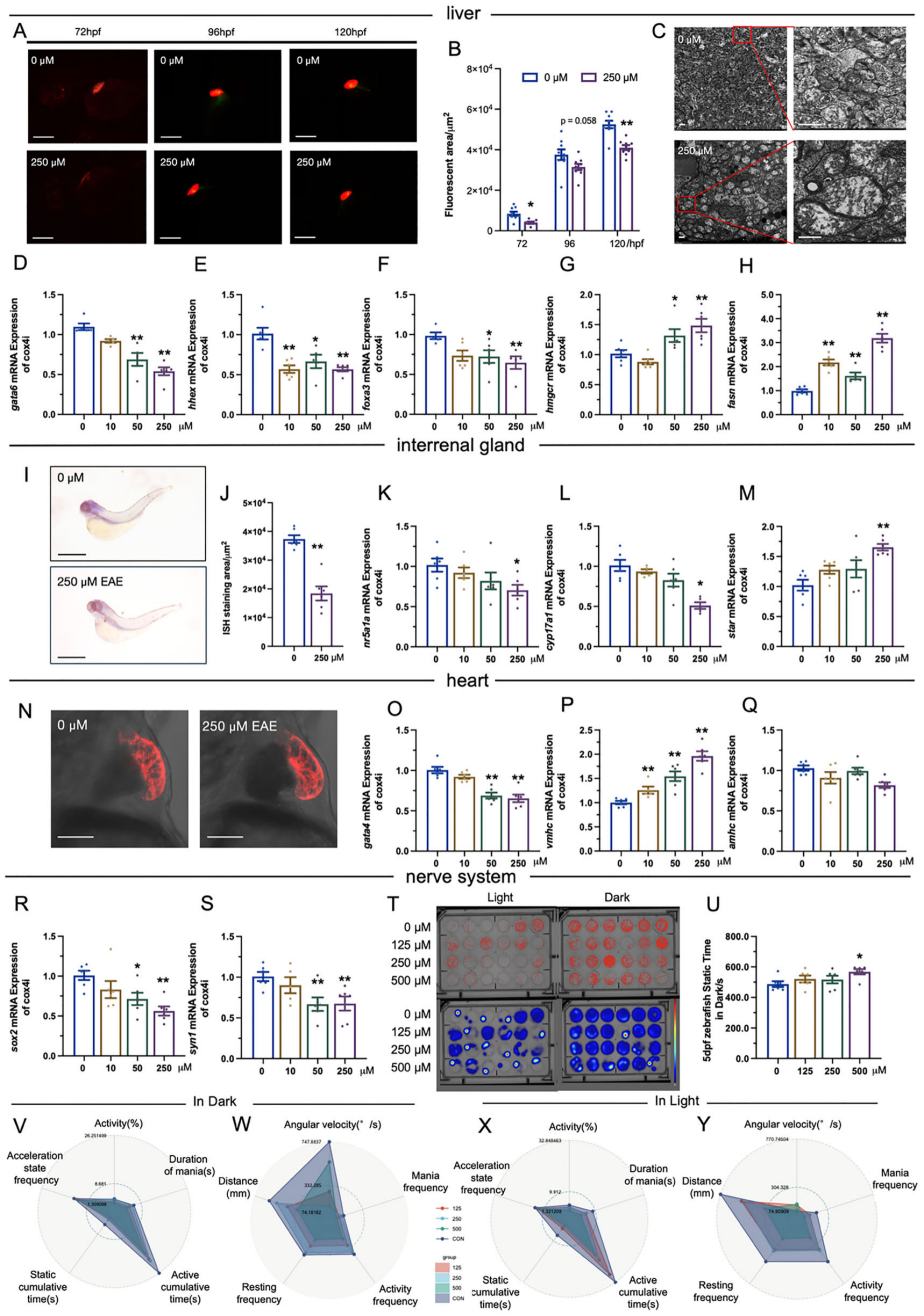
Multi‐organ morphological and functional alterations in 120 hpf zebrafish larvae following EAE. Liver: (A) Tg(fabp10a:mCherry; ela:eGFP) liver morphology. Scale bar, 500 µm. (B) Fluorescent area (n = 9). (C) TEM of hepatic mitochondria. Scale bar, 1 µm. (D–H) Relative gene expression of *gata6, hhex, foxa3, fasn* and *hmgcr*. Interrenal gland: (I) Representative *nr5a1a* ISH images. Scale bar, 500 µm. (J) Staining area of *nr5a1a* ISH. (K–M) Relative gene expression of *nr5a1a, cyp17a1*, and *star*. Heart: (N) Tg(vMHC: ala) heart morphology. Scale bar, 100 µm. (O–Q) Relative gene expression of *gata4*, *vmhc*, and *amhc* expression. Brain: (R,S) Relative gene expression of *sox2* and *syn*. (T) Representative tracks in the light‐dark test. (U) Static time under dark conditions. (V–Y) Radar plots of behavioral metrics under dark and light conditions. Data are mean ± S.E.M. n = 6 unless indicated. ^*^ *P<*0.05, ^**^ *P*<0.01 *vs*. control. Unpaired *t*‐test for two independent groups, and one‐way analysis of variance (ANOVA), followed by Tukey's multiple comparison test for more than two independent samples. EAE, Embryonic Amoxicillin Exposure.

To further evaluate the effects of EAE on multi‐organ development, we examined morphological and functional indicators in the liver, interrenal gland, heart, and nervous system of zebrafish larvae. The results showed that EAE caused delayed liver development (Figure 2A), with significantly reduced hepatic fluorescence area (*P*<0.05, *P*<0.01, Figure 2B). Transmission electron microscopy revealed mitochondrial swelling, vacuolization, and loss of cristae in hepatocytes (Figure 2C). Expression of hepatic progenitor differentiation genes *gata6, hhex*, and *foxa3* was significantly decreased (*P*<0.05, *P*<0.01, Figure 2D–F), whereas fatty acid synthesis gene fasn and cholesterol metabolism gene hmgcr were significantly upregulated (*P*<0.05, *P*<0.01, Figure 2G,H). In the interrenal gland, the in situ hybridization area of *nr5a1a* was reduced (*P*<0.01, Figure 2I,J). Expression of differentiation‐related genes *nr5a1a* and *cyp17a1* decreased in a concentration‐dependent manner (*P*<0.05, Figure 2K,L), while the cholesterol transport gene *star* was significantly upregulated (*P*<0.01, Figure 2M). Although no obvious morphological changes were observed in the heart (Figure 2N), the cardiomyocardial differentiation gene *gata4* was significantly decreased with increasing EAE concentrations (*P*<0.01, Figure 2O), while the ventricular marker gene *vmhc* was significantly upregulated (*P*<0.01, Figure 2P) and the atrial marker *amhc* remained unchanged (Figure 2Q). In the brain, expression of the neural stem cell differentiation gene sox2 and the synaptic protein gene syn1 was significantly decreased following EAE (*P*<0.05, *P*<0.01, Figure 2R,S). Behavioral analyses further showed altered locomotor trajectories in EAE‐treated larvae (Figure 2T), with significantly increased immobility time under dark conditions (*P*<0.05, Figure 2U). Additional locomotor parameters also exhibited dose‐dependent alterations (Figure 2V–Y). Additionally, no significant alterations in organ‐specific gene expression or functional markers were observed in lower‐dose (2 µm) exposure (Figure S2A).

Collectively, these findings indicate that EAE disrupts multi‐organ development in zebrafish larvae, leading to morphological alterations and impaired cellular differentiation across multiple tissues.

### 2.3 Upregulation of lncRNA PCALRx Mediates EAE‐Induced Multi‐Organ Developmental Defects in Zebrafish Larvae

**FIGURE 3:**
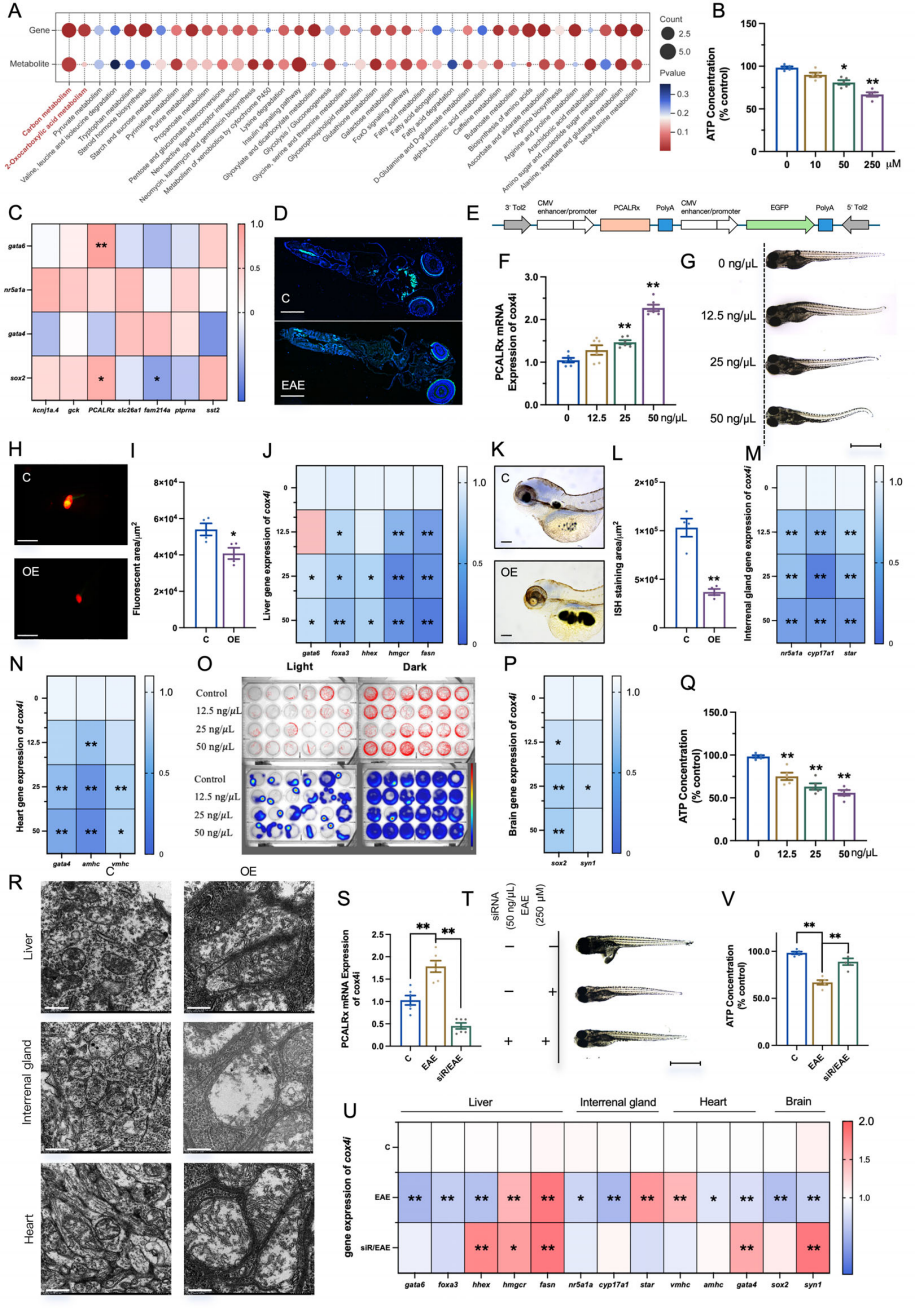
High expression of LncRNA PCALRx mediates multi‐organ developmental damage in zebrafish larvae caused by EAE. (A) Integrated multi‐omics KEGG enrichment. (B) Whole‐body ATP of 120 h EAE (n = 5). (C) Correlation of differentially expressed genes with organ functional markers. (D) PCALRx RNA‐FISH staining after EAE. Scale bar, 300 µm. **PCALRx overexpression**: (E) Tol2‐based PCALRx overexpression construct. (F) Expression validation. (G) Representative bright‐field larvae imgaes. Scale bar, 1000 µm. (H) Liver morphology of Tg(fabp10a: mCherry; ela: eGFP). Scale bar, 500 µm. (I) Fluorescent area (n = 4). (J) Relative hepatic gene expression heat map. (K) Interrenal gland *nr5a1a* ISH. Scale bar, 200 µm. (L) Interrenal gland ISH staining area (n = 4). (M) Relative interrenal gland gene expression heat map. (N) Relative cardiac gene expression heat map. (O) Representative tracks in the light/dark test. (P) Relative brain gene heat map. (Q) Whole‐body ATP (n = 5). (R) TEM of mitochondria. Scale bar, 500 nm. **PCALRx knockdown**: (S) knockdown efficiency. (T) Representative larvae. Scale bar, 1000 µm. (U) Relative organ marker expression heat map. (V) Whole‐body ATP (n = 5). Data are mean ± S.E.M. n = 6 unless indicated. ^*^ *P<*0.05, ^**^ *P*<0.01 *vs*. control. Unpaired *t*‐test for two independent groups, and one‐way analysis of variance (ANOVA), followed by Tukey's multiple comparison test for more than two independent samples. C, control. OE, PCALRx over‐expression. EAE, embryonic amoxicillin exposure. siR/EAE, PCALRx small interfering RNA + embryonic amoxicillin exposure.

To investigate the mechanisms underlying EAE‐induced multi‐organ developmental and differentiation impairments, we performed metabolomic (Figure S3A–C) and transcriptomic (Figure S3D,E) analyses of EAE‐treated zebrafish larvae, followed by integrated multi‐omics analysis. Spearman correlation and network analyses (Figure S3F–I) revealed coordinated gene–metabolite associations involving carbon metabolism and TCA‐cycle intermediates, while CCA further supported transcriptome–metabolome coupling. A total of 39 co‐annotated KEGG pathways were identified (Figure S3J), among which 2‐oxocarboxylic acid metabolism and carbon metabolism pathways were significantly altered (Figure 3A), suggesting that EAE may impair organ development through disruption of mitochondrial energy metabolism. Whole‐body ATP measurements at 120 hpf revealed a significant, dose‐dependent reduction in total ATP levels following EAE (*P*<0.05, *P*<0.01, Figure 3B), further supporting a link between mitochondrial dysfunction and multi‐organ developmental impairment.

Correlation analysis between significantly altered transcriptomic genes and previously identified organ‐specific functional genes revealed that lncRNA PCALRx (*URS0000A7E830*) was highly correlated with multi‐organ functional gene changes (*P*<0.05, *P*<0.01, Figure 3C; Figure S4), suggesting that PCALRx may represent a common target mediating amoxicillin‐induced developmental toxicity. Whole‐mount RNA‐FISH qualitatively showed an increase in PCALRx signal, whereas qPCR analysis quantitatively demonstrated significant concentration‐dependent upregulation of PCALRx following EAE (*P*<0.01, Figure 3D; *P*<0.05, *P*<0.01, Figure S3K,L). Additionally, PCALRx expression showed significant correlations with key TCA metabolites, including citrate, malate, and aspartate (Figure S3M–O), which further supported a link between PCALRx upregulation and mitochondrial metabolic disruption under EAE conditions.

To determine whether increased PCALRx expression was sufficient to induce the observed developmental phenotypes, we constructed a Tol2‐based PCALRx overexpression plasmid carrying an independent EGFP reporter cassette (Figure 3E, Table S2) and microinjected it into one‐cell‐stage zebrafish embryos to perform a gain‐of‐function experiment. PCALRx and EGFP were expressed from separate transcriptional cassettes. Overexpression significantly increased PCALRx levels at 120 hpf in overall tissues (*P*<0.01, Figure 3F; Figure S5A) and recapitulated phenotypes observed in the EAE group. We used RNA‐FISH results as qualitative supporting evidence of increased PCALRx signal. Specifically, increased plasmid dosage led to higher mortality (Figure S5B), as well as concentration‐dependent reductions in body length and head length (Figure 3G; *P*<0.05, *P*<0.01, Figure S5D,E). Organ‐specific analyses showed delayed liver development in morphology (*P*<0.05, Figure 3H,I), with decreased expression of *gata6, foxa3, hhex, hmgcr* and *fasn* (*P*<0.05, *P*<0.01, Figure 3J; Figure S5F–J). In the interrenal gland, the in situ hybridization area of *nr5a1a* was reduced (*P<*0.01, Figure 3K,L), and *nr5a1a, cyp17a1*, and *star* expression levels were significantly decreased (*P*<0.01, Figure 3M; Figure S5K–M). Cardiac markers *gata4, amhc*, and *vmhc* were also significantly reduced (*P<*0.01, Figure 3N; Figure S5N–P). Behavioral alterations were observed, including changes in locomotor activity (Figure 3O; Figure S5S,T), accompanied by decreased brain expression of *sox2* and *syn1* (*P*<0.05, *P*<0.01, Figure 3P; Figure S5Q,R). Moreover, whole‐body ATP levels declined in a dose‐dependent manner following PCALRx overexpression (*P*<0.01, Figure 3Q), and mitochondrial swelling was observed across multiple organs (Figure 3R).

Conversely, knockdown of PCALRx under EAE conditions via siRNA microinjection significantly reduced PCALRx expression at 120 hpf (*P<*0.01, Figure 3S). Bright‐field imaging showed restoration of overall morphology and body length (Figure 3T; Figure S5U). Multi‐organ functional markers returned toward control levels (*P*<0.05, *P*<0.01, Figure 3U; Figure S5V–h), and whole‐body ATP content was significantly recovered (*P*<0.01, Figure 3V). The behavioral trajectory after knockdown was also restored to normal (Figure S5i).

In summary, these findings indicate that EAE upregulates PCALRx expression, which suppresses mitochondrial energy metabolism, leading to impaired multi‐organ differentiation and reduced expression of organ‐specific developmental genes in zebrafish larvae.

### 2.4 Identification of Pyruvate Carboxylase as a PCALRx‐Associated Candidate Protein in EAE‐Induced Multi‐Organ Impairment

**FIGURE 4:**
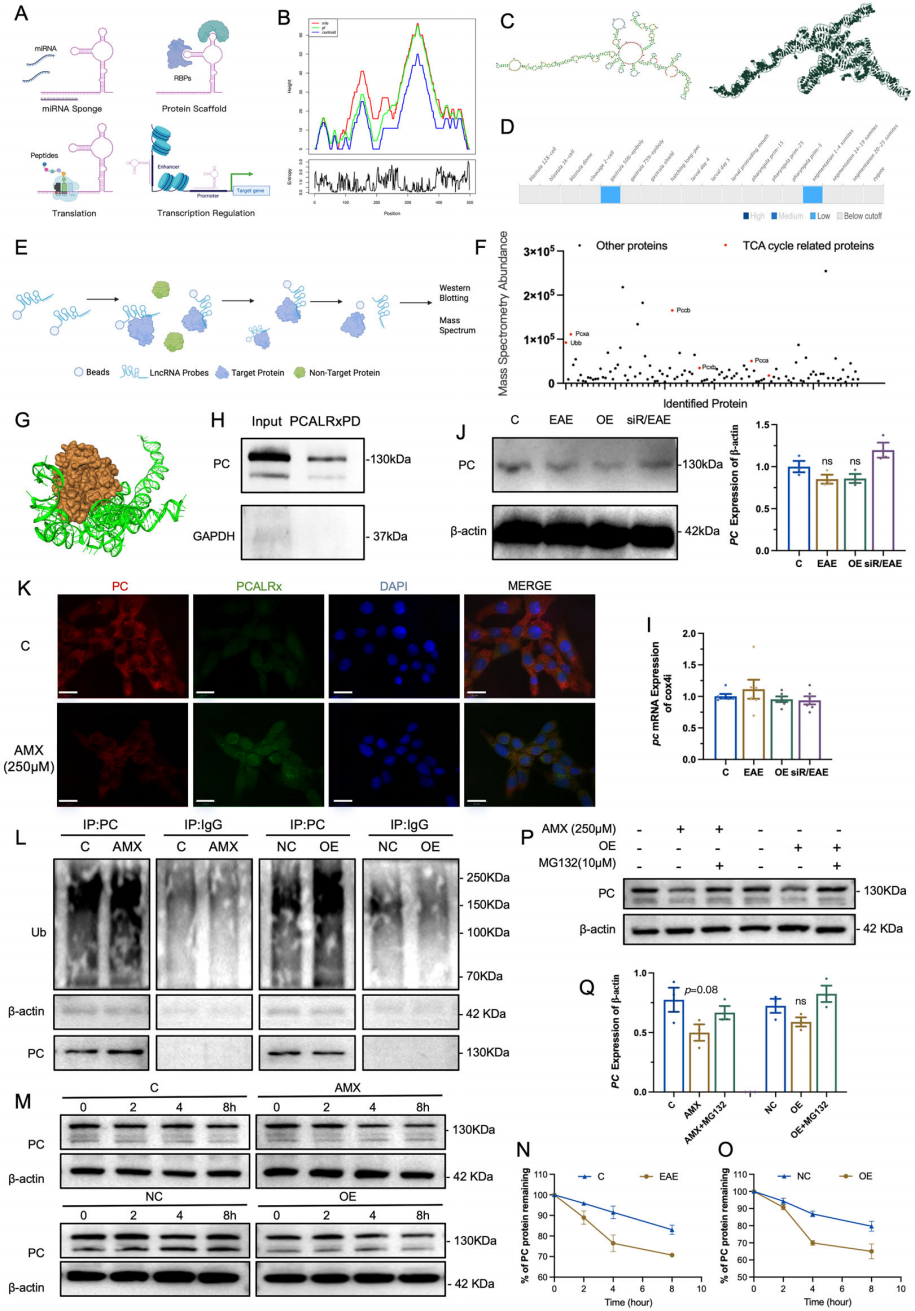
Interaction between lncRNA PCALRx and pyruvate carboxylase. (A) Schematic of potential lncRNA mechanisms. (B) Predicted thermodynamic ensemble mountain plot of PCALRx. (C) Secondary/3D structure of PCALRx. (D) Expression period of PCALRx. (E) RNA–protein pull‐down workflow. (F) Identified proteins by MS. (G) Docking model of PCALRx with pyruvate carboxylase (PC). (H) PC in pull‐down eluates (western blot). (I) Relative *pc* mRNA expression (n = 6). (J) PC protein across western blot groups (n = 3). (K) Immunofluorescence for PC (red), PCALRx RNA‐FISH (green), and DAPI (blue) in ZF4 cells. Scale bar, 10 µm. (L) Immunoprecipitation of ZF4 cells PC followed by immunoblotting for ubiquitin. IgG was used as the negative immunoprecipitation control. (M) Cycloheximide chase assay of ZF4 cells PC protein at 0, 2, 4, and 8 h after CHX treatment in control, EAE‐treated, NC, and PCALRx‐overexpressing groups. (N, O) Quantification of PC protein levels over time in the CHX chase assay (n = 3). (P) Western blot analysis of ZF4 cells PC protein levels with or without MG132 treatment. (Q) Quantification of pc mRNA expression(n = 3). Data are mean ± S.E.M. ^*^ *P<*0.05, ^**^ *P*<0.01 *vs*. control. Unpaired *t*‐test for two independent groups, and one‐way analysis of variance (ANOVA), followed by Tukey's multiple comparison test for more than two independent samples. C, control. EAE, embryonic amoxicillin exposure. OE, PCALRx over‐expression. siR/EAE, PCALRx small interfering RNA + embryonic amoxicillin exposure.

To further elucidate the mechanism by which PCALRx disrupts mitochondrial function and impairs multi‐organ differentiation, we investigated its potential RNA–protein interactions. LncRNAs exert biological functions through diverse mechanisms, including RNA–protein binding (Figure 4A). Secondary and tertiary RNA structure predictions indicated that PCALRx contains two stem–loop structures (Figure 4B,C). No evidence suggests that PCALRx encodes peptides or small proteins, and its expression is not detected during later developmental stages under normal conditions (Figure 4D), supporting its classification as a non‐coding regulatory RNA.

RNA pulldown followed by LC–MS/MS analysis [36, 40, 41] was performed for qualitative analysis to identify potential proteins interacting with PCALRx (Figure 4E). The results revealed that PCALRx may physically associate with several key tricarboxylic acid (TCA) cycle enzymes, including pyruvate carboxylase (Pcxa, Pcxb) and propionyl‐CoA carboxylase (Pcca, Pccb) (Figure 4, Figure S6A). Molecular docking analysis predicted a potential association between PCALRx and PC, and western blot further confirmed the enrichment of PC in the PCALRx pull‐down fraction (Figure 4G,H). These findings support a potential association between PCALRx and PC under the RNA pull‐down conditions.

We next examined PC mRNA and protein levels in EAE‐treated larvae, PCALRx‐overexpressing larvae, and PCALRx knockdown larvae. PC mRNA expression showed no significant changes (Figure 4I), whereas western blot analysis indicated a decreasing trend in PC protein levels under EAE and PCALRx overexpression conditions, with partial recovery following siRNA‐mediated knockdown (Figure 4J). Immunofluorescence and RNA‐FISH staining in ZF4 cells similarly demonstrated reduced PC protein levels and increased PCALRx levels following 250 µm EAE treatment (Figure 4K; Figure S6B). These findings suggest that PCALRx may reduce PC protein abundance through post‐transcriptional regulation, potentially involving protein destabilization or degradation.

To further elucidate the underlying mechanism, we assessed global protein ubiquitination levels in amoxicillin‐treated and PCALRx‐overexpressing ZF4 cell lines. The results showed that overall ubiquitination levels were increased in PCALRx‐overexpressing cells compared with the NC group (Figure S6C). Co‐immunoprecipitation assays further demonstrated that ubiquitination of PC protein was significantly elevated in PCALRx‐overexpressing larvae (Figure 4L), suggesting that PCALRx may promote PC ubiquitination. Next, we evaluated PC protein stability using a cycloheximide (CHX) chase assay in ZF4 cells transfected with a PCALRx overexpression plasmid. Cells were treated with 50 µg mL^−1^ CHX and collected at 0, 2, 4, and 8 h for Western blot analysis. The results showed that PC protein degradation was markedly accelerated in the PCALRx overexpression group compared with the NC group (Figure 4M–O), indicating reduced PC protein stability. Furthermore, we treated PCALRx over‐expressing ZF4 cells with 10 µm MG132 for 8 h. Western blot analysis revealed that MG132 treatment significantly rescued the reduction of PC protein induced by PCALRx overexpression (Figure 4P,Q), suggesting that the decrease in PC protein is mediated through the proteasome pathway.

Taken together, these results indicate that PCALRx directly interacts and promotes PC protein ubiquitination and facilitates its degradation via the ubiquitin–proteasome system, thereby reducing PC protein abundance.

### 2.5 Vitamin B1 Mitigates EAE‐Induced Multi‐Organ Morphological and Functional Developmental Defects

**FIGURE 5:**
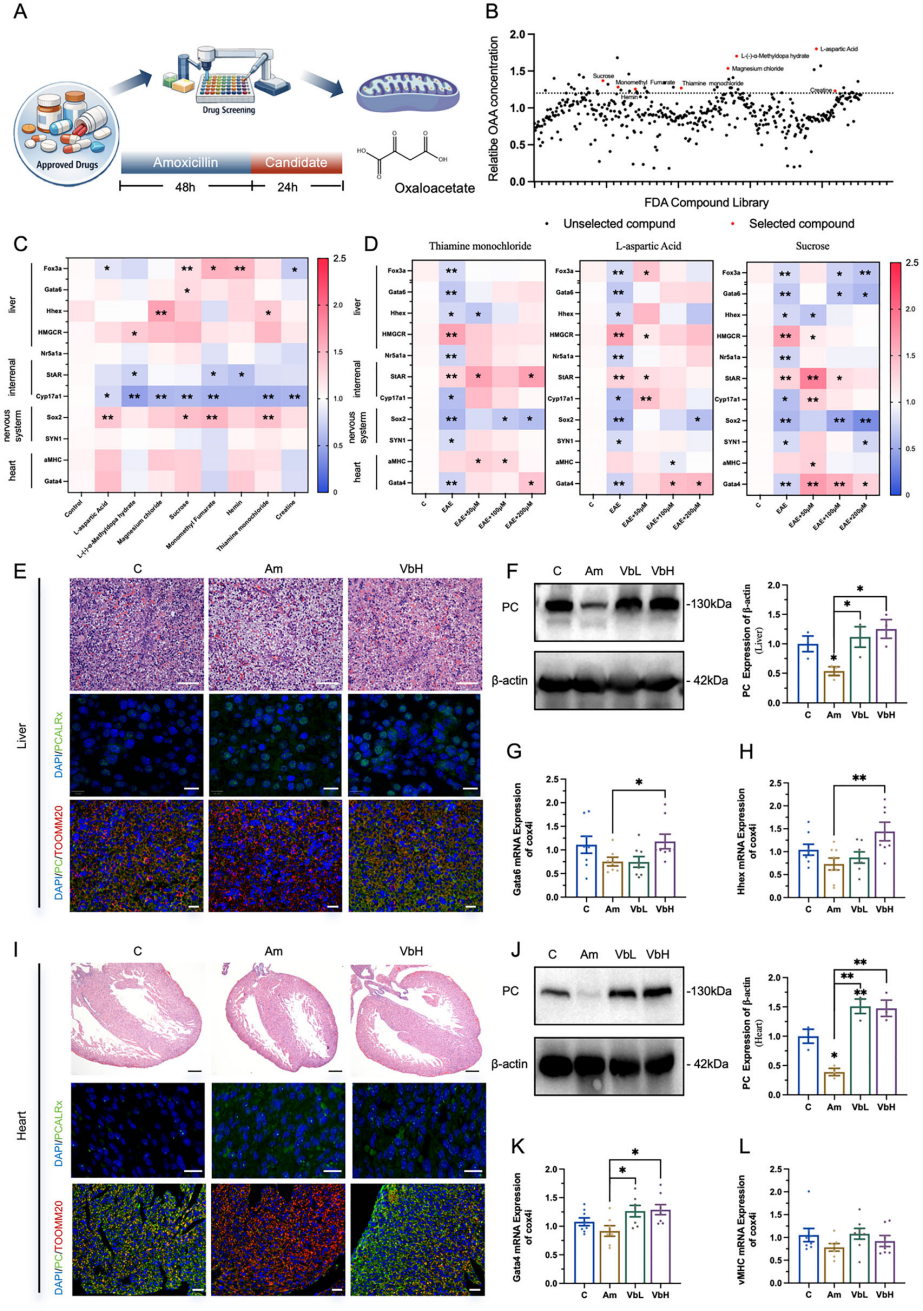
Drug repurposing strategy to mitigate EAE‐induced developmental alterations. (A) Drug‐repurposing workflow. (B) Oxaloacetate fold change in amoxicillin‐pretreated ZF4 cells after exposure to FDA‐approved compounds. (C) Heat map of organ markers in larvae co‐exposed to amoxicillin and selected compounds. (D) Dose responses for vitamin B1, L‐aspartic acid, and sucrose. (E) H&E, PCALRx FISH (scale bar, 10 µm), and PC (green) / TOMM20 (red) immunofluorescence in GD20 mice liver. Scale bar, 50 µm. (F) PC protein expression in mice liver. (western blot; n = 3). (G,H) Hepatic Gata6 and Hhex relative mRNA expression. (I) H&E (scale bar, 200 µm), PCALRx FISH (scale bar, 20 µm), and PC (green) / TOMM20 (red) immunofluorescence (scale bar, 50 µm) in GD20 mice heart. (J) PC protein expression in mice heart (western blot; n = 3). (K,L) Cardiac Gata4 and vMHC relative mRNA expression. Data are mean ± S.E.M. n = 8 unless indicated. ^*^ *P<*0.05, ^**^ *P*<0.01 *vs*. control. Unpaired *t*‐test for two independent groups, and one‐way analysis of variance (ANOVA), followed by Tukey’ s multiple comparison test for more than two independent samples. C, control; Am, prenatal amoxicillin exposure; VbL/VbH, Am + low/high vitamin B1. GD, gestational day.

To identify compounds capable of alleviating EAE‐induced developmental toxicity, we employed a drug‐repurposing strategy to screen approved drugs for protective effects against amoxicillin‐induced developmental impairment. Given that EAE was shown to reduce PC protein levels potentially through PCALRx‐mediated regulation, ZF4 cells were pretreated with amoxicillin for 48 h, followed by 24 h exposure to candidate compounds from the FDA database. The ability of these compounds to restore downstream metabolites of PC was assessed (Figure 5A). Based on pharmacological properties and dose‐response validation in vitro, vitamin B1, L‐aspartate, and sucrose were identified as compounds that enhanced PC activity (Figure 5B; Figure S7A–H).

In vivo validation in zebrafish larvae demonstrated that eight screened compounds partially rescued EAE‐induced multi‐organ functional impairment (*P*<0.05, *P*<0.01, Figure 5C), with improvements in body length, head length, and overall morphology (Figure S7I–Y). Among these, vitamin B1 exhibited the most robust restorative effect, approaching control levels more closely than L‐aspartate or sucrose (*P*<0.05, *P*<0.01, Figure 5D).

To evaluate cross‐species consistency, vitamin B1 intervention was further examined in a mouse model of prenatal amoxicillin exposure [29, 42, 43, 44]. Although overall pregnancy outcomes were not significantly altered (Figure S8A–D), offspring from prenatal amoxicillin‐exposed mice exhibited hepatic vacuolization, which was alleviated following vitamin B1 supplementation (Figure 5E, 1^st^ row). Given that the functional conservation of lncRNAs may be mediated by short conserved fragments, structural motifs, or functional domains rather than full‐length sequence conservation, we further examined PCALRx‐related lncRNA expression in multiple mouse organs. Although the BLAST‐based analysis indicated limited primary‐sequence conservation between PCALRx and other species’ genomes (Figure S8E), FISH analysis in mouse liver still showed increased PCALRx expression under amoxicillin exposure (Figure 5E, 2^nd^ row; Figure *P*<0.01, S9A,E), while PC protein levels were significantly reduced (Figure 5E, 3^rd^ row; *P*<0.01, Figure S9F); vitamin B1 intervention restored PC protein abundance (*P*<0.05, Figure 5F). Hepatic functional genes, including Gata6 and Hhex, were also partially restored (*P*<0.05, *P*<0.01, Figure 5G,H; Figure S8F–J). Cardiac histological analysis revealed ventricular wall thinning following prenatal amoxicillin exposure, accompanied by increased PCALRx expression and decreased PC (Figure 5I; *P*<0.05, Figure S9B,G). Vitamin B1 supplementation restored PC protein levels in cardiac tissue (*P*<0.05, *P*<0.01, Figure 5J), and cardiac functional gene expression trends moved toward control levels (*P*<0.05, Figure 5K,L; Figure S8K–M). Similar restorative trends were observed in adrenal and brain‐related functional genes (*P*<0.05, *P*<0.01, Figure S8N–R), with adrenal tissues showing amoxicillin‐induced upregulation of PCALRx and downregulation of PC protein, both of which were reversed following vitamin B1 supplementation (*P*<0.05, *P*<0.01, Figure S9C,D,I,J). Moreover, PC knockdown in zebrafish larvae attenuated vitamin B1‐mediated protection against EAE‐induced multi‐organ developmental impairment, suggesting that this rescue effect is at least partly dependent on PC protein abundance (Figure S9K,L).

Collectively, these findings indicate that vitamin B1 mitigates PCALRx‐related mitochondrial dysfunction and multi‐organ developmental impairment induced by amoxicillin in zebrafish larvae and demonstrates consistent protective effects in a mammalian model.

## 3 Discussion

### 3.1 Advantages of Zebrafish for Multi‐Organ Toxicity Assessment and Establishment of the EAE Model

Zebrafish are widely used in environmental toxicology due to external embryonic development, optical transparency, well‐defined organogenesis timelines, and substantial genetic homology with humans (∼71%) [45]. High conservation of developmental regulatory pathways and metabolic networks enables mechanistic evaluation of chemical‐induced developmental perturbations [46, 47]. Compared with rodent models, zebrafish embryos complete major organ formation within a short time window, facilitating rapid and integrated assessment of multi‐organ toxicity. Importantly, embryonic zebrafish exhibit heightened sensitivity to metabolic disturbances, making them particularly suitable for evaluating energy metabolism–related developmental toxicity [48, 49, 50, 51, 52]. Importantly, embryonic and larval zebrafish often exhibit toxicological responses distinct from those observed in adult fish [45], reflecting developmental stage–specific vulnerability. This characteristic makes embryonic zebrafish a suitable model for identifying early‐life sensitive windows and sublethal molecular alterations that precede overt morphological defects, which are increasingly recognized as critical endpoints in environmental risk assessment.

To evaluate the impact of embryonic exposure on multi‐organ development, we used an EAE model in zebrafish. In previous reports, the concentration of amoxicillin detected in the maternal blood serum was 2.18 ± 1.30 µg g^−1^ (2.41–9.52 µm) [53]. While human clinical dosing (750–1000 mg day^−1^) corresponds to an estimated zebrafish‐equivalent exposure range of 342–456 µm based on body surface area–normalized conversion [54]. Considering previously reported toxic thresholds in zebrafish [55] and the experimentally determined LC_50_ value (344 µm) in this study, exposure concentrations of 0, 2, 10, 50, and 250 µm were selected. This concentration range represents a limited and controlled toxicological extrapolation. Mechanistic investigations at 250 µm enabled a systematic assessment of dose‐dependent developmental, metabolic, and molecular alterations while avoiding excessive nonspecific lethality.

### 3.2 Dose–Response Characteristics and Organ Sensitivity to EAE

EAE produced clear dose‐ and time‐dependent developmental toxicity in zebrafish larvae, manifested by increased mortality and malformation rates, along with significant reductions in body and head length. These findings confirm that amoxicillin can disrupt early developmental processes and demonstrate the high sensitivity of zebrafish embryos as a model for detecting environmentally relevant toxic effects. The consistent dose‐ and time‐dependent alterations in developmental phenotypes, organ differentiation markers, and metabolic pathways indicate that amoxicillin‐induced developmental disruption follows a coherent toxicological pattern rather than resulting from nonspecific toxicity. Notably, significant alterations in multi‐organ differentiation–related gene expression were observed under sublethal exposure conditions, indicating that molecular perturbations preceded overt morphological abnormalities. This pattern is consistent with the toxicological principle that sublethal molecular and metabolic disturbances often occur before structural defects become apparent [56].

Organ‐specific analyses revealed differential sensitivity among tissues. Such heterogeneity likely reflects differences in developmental timing, metabolic demand, and energy dependence. Early neural development is highly reliant on mitochondrial function and ATP supply [57], making it particularly vulnerable to metabolic disruption. Similarly, the interrenal gland and liver serve as central hubs for steroidogenesis and metabolic regulation [58, 59], processes that require tightly regulated mitochondrial energy metabolism. In contrast, cardiac morphogenesis, although initiated early [60], may be developmentally buffered to preserve essential circulatory function. This pattern aligns with developmental plasticity concepts, including the “thrifty phenotype” hypothesis within the DOHaD framework [61], which proposes that embryos may prioritize critical organ systems under stress conditions. In addition, previous studies have reported that zebrafish exposed to amoxicillin after 12 hpf exhibit only limited liver alterations [31], suggesting that early embryonic environmental conditions may play a critical role in shaping multi‐organ developmental outcomes.

At the 10 µm concentration group, no significant organ‐level impairment was detected. This concentration is comparable to the reported concentration in livestock wastewater (up to 3344.4 µg L^−1^) [62], but exceeds most reported environmental levels in surface waters [63, 64, 65, 66]. However, amoxicillin undergoes environmental transformation, and wastewater treatment processes do not completely remove its degradation products. The clear concentration‐ and duration‐dependent increases in mortality, developmental abnormalities, growth impairment, ATP reduction, and organ‐specific molecular alterations further support the biological consistency of the exposure paradigm and indicate that amoxicillin‐induced developmental toxicity exhibits cumulative characteristics with increasing exposure concentration and duration. Therefore, the long‐term ecological and developmental consequences resulting from long‐term low‐dose exposure to amoxicillin and its metabolites remain to be confirmed [67, 68]. Future studies should address chronic exposure scenarios to better reflect environmentally realistic conditions.

### 3.3 LncRNA as a Convergent Regulator of pc Suppression in EAE‐Induced Multi‐Organ Toxicity

Mechanistically, Multi‐omics analyses showed that EAE disrupted mitochondrial energy metabolism in zebrafish larvae, including affecting TCA cycle–related metabolites and pathways, suggesting that metabolic disequilibrium represents a central mechanism linking amoxicillin exposure to multi‐organ differentiation defects. Long non‐coding RNAs (lncRNAs) are increasingly recognized as regulators of metabolic homeostasis, capable of modulating enzymatic activity or mitochondrial‐associated proteins to influence cellular energy status and differentiation programs [69, 70, 71, 72]. In this study, transcriptomic profiling and functional screening identified PCALRx as a candidate lncRNA strongly correlated with multi‐organ differentiation impairment. PCALRx was significantly upregulated following EAE. Its overexpression dose‐dependently suppressed differentiation marker genes across multiple organs, whereas knockdown partially rescued EAE‐induced developmental abnormalities, supporting a necessary regulatory role in amoxicillin‐associated toxicity.

PC is a key anaplerotic enzyme replenishing TCA cycle intermediates, and its dysfunction is known to impair mitochondrial metabolism, reduce ATP production, and disrupt tissue differentiation, particularly during development [73]. In this study, PC protein levels of EAE embryos were reduced without significant changes at the mRNA level, accompanied by decreased ATP content. Meanwhile, RNA pull‐down coupled with mass spectrometry identified PC as a candidate PCALRx‐associated protein, and immunoblotting confirmed the enrichment of PC in the PCALRx pull‐down fraction. Together with the increased ubiquitination and accelerated proteasome‐dependent degradation of PC following PCALRx overexpression, these results suggest that PCALRx may participate in the regulation of PC protein stability, thereby contributing to mitochondrial metabolic suppression. However, whether PCALRx directly binds to PC in vivo remains to be established. Interestingly, although PCALRx lacks annotation in current cross‐species lncRNA databases and shows limited primary sequence conservation, fluorescence in situ hybridization indicated its expression in multiple mouse organs, with similar upregulation patterns under prenatal amoxicillin exposure. This observation suggests PCALRx's potential functional conservation at the regulatory level, supporting the hypothesis that lncRNA‐mediated modulation of PC may represent a conserved metabolic vulnerability node in amoxicillin‐related developmental toxicity. Future studies should focus on extending these findings to human‐relevant systems to further evaluate the conservation and translational significance of the PCALRx‐related amoxicillin–PC–mitochondrial dysfunction axis. Human cell‐based models, including hepatocyte, adrenal, and neural progenitor cell lines, may provide valuable platforms to investigate whether modulation of PCALRx orthologous transcripts or functionally analogous lncRNAs influences PC stability, ubiquitination dynamics, and mitochondrial energy metabolism.

### 3.4 Drug Repurposing Accelerates Translational Application in Developmental Toxicology

This study applied a drug repurposing strategy targeting PC activity and ABUC identified as a central event in amoxicillin‐induced developmental toxicity. This approach enables rapid identification of candidate metabolic modulators and shortens translational timelines by established pharmacokinetic profiles and safety records of approved compounds [38, 39]. As a necessary cofactor for mitochondrial enzyme complexes, vitamin B1 is generally regarded as having a positive promoting effect on neural development [74, 75, 76]. In this study, we proved that vitamin B1 is the most effective candidate for promoting multi‐organ development through sequential screening in ZF4 cells and whole‐animal models. Vitamin B1 could restore multi‐organ differentiation defects in zebrafish and showed consistent protective trends in a prenatal amoxicillin exposure mouse model. Notably, PC was identified as a convergent toxicity target in both zebrafish and mouse models, and vitamin B1 intervention consistently restored PC protein levels and downstream functional markers. These findings suggest that PC may represent an evolutionarily conserved metabolic vulnerability node underlying amoxicillin‐associated developmental toxicity. Increasing evidence indicates that metabolic reprogramming is a central feature of developmental toxicology, and targeting conserved mitochondrial pathways is emerging as a rational intervention strategy [77, 78]. The vitamin B1 intervention results suggest a potential public‐health hypothesis, particularly under unavoidable antibiotic exposure during pregnancy. As vitamin B1‐containing prenatal multivitamins are widely used, adequate thiamine may help support mitochondrial metabolic resilience. However, this study provides preclinical evidence rather than direct clinical validation. Thus, these findings should not be interpreted as a recommendation for additional or high‐dose vitamin B1 supplementation without medical guidance, nor do they alter guideline‐based amoxicillin use during pregnancy. Clinical translation will require further evaluation of dose, timing, maternal–fetal pharmacokinetics, safety, and efficacy in prospective studies.

Collectively, these data support the feasibility of drug repurposing as a mechanistically guided approach for identifying metabolic protectants and provide experimental evidence for targeting conserved mitochondrial nodes in developmental toxicity contexts [79].

### 3.5 Limitations

Despite integrating multi‐organ phenotyping, multi‐omics profiling, and mechanistic validation, several limitations warrant consideration. First, the temporal scope of this study was limited to embryonic and early developmental stages. Long‐term consequences of amoxicillin exposure on growth trajectories, metabolic homeostasis, and behavioral phenotypes were not assessed. Given that early‐life metabolic perturbations may predispose individuals to later‐life dysfunction, longitudinal studies incorporating chronic or delayed outcome assessments are necessary. Second, because zebrafish larvae at 120 hpf are too small for reliable dissection and isolation of individual organs, all qRT‐PCR analyses were performed using total RNA extracted from whole larvae. Consequently, although organ‐enriched functional markers could be assessed at the whole‐larva level, precise organ‐specific quantification of PCALRx was not feasible. Future studies using later developmental stages, tissue‐specific reporter models, or spatial and single‐cell transcriptomic approaches will be required to define the organ‐ and cell‐specific distribution of PCALRx. At the mechanistic level, although PCALRx was shown to directly interact with PC and modulate its protein abundance, Future studies should further define the functional domains of PCALRx to clarify the structural basis of its regulatory activity and evaluate the broader applicability of targeting lncRNA‐mediated mitochondrial pathways in developmental toxicology.

## 4 Conclusion

This study demonstrates that EAE induces multi‐organ developmental toxicity in zebrafish larvae, with organ‐specific sensitivity. At the molecular level, we identified and characterized a previously unannotated lncRNA, PCALRx (URS0000A7E830), which was significantly upregulated following EAE. Functional analyses indicate that this lncRNA directly interacts with mitochondrial PC, reduces mitochondrial function, and contributes to systemic energy imbalance and impaired organ differentiation. Using a mechanistically guided drug repurposing strategy, vitamin B1 was identified as an effective metabolic modulator capable of mitigating EAE‐induced developmental defects. The protective effect was consistently observed in both zebrafish larvae and prenatal mouse exposure models, supporting the role of PC as a conserved metabolic vulnerability node across species. Overall, these findings reveal a lncRNA‐driven mitochondrial metabolic mechanism underlying amoxicillin‐related developmental toxicity and provide experimental evidence supporting metabolism‐based intervention strategies.

## 5 Experimental Section

### 5.1 Zebrafish Husbandry and Amoxicillin Treatment

Wild‐type AB adult zebrafish were obtained from the Zebrafish Center at the Institute of Hydrobiology, Chinese Academy of Sciences, and were maintained under standard conditions (ambient temperature 28 ± 0.5°C, 14 h light/10 h dark cycle) at the Zebrafish Core Facility, Wuhan University School of Medicine. The zebrafish lines used in this study included wild‐type AB strain and transgenic lines Tg(vmhc: ala) and Tg(fabp10a:mCherry; ela:eGFP). Male and female adult zebrafish (1:1 ratio) were placed in breeding tanks with movable partitions for overnight acclimation. The partition was removed at 9:00 AM the following day, and embryos were collected 30 min later. Embryo mortality and abnormal rates were recorded daily at 9:00 AM. Mortality rate was calculated as the number of dead embryos divided by the total number of embryos on the previous day, multiplied by 100%. Developmental abnormalities were assessed at 24, 48, 72, 96, and 120 hpf under a stereomicroscope. Embryos or larvae were scored as abnormal if they displayed one or more of the following phenotypes: pericardial edema, spinal curvature, tail malformation, delayed hatching, craniofacial abnormality, impaired pigmentation, or severe developmental delay. The abnormal rate was calculated as the number of abnormal embryos or larvae divided by the total number of live embryos or larvae examined in each biological replicate, multiplied by 100%. Each biological replicate consisted of a pool of 20 embryos, and all experimental groups were performed with at least three independent replicates.

All exposure protocols were performed strictly in accordance with IACUC guidelines to minimize zebrafish pain. Embryos were exposed to a series of amoxicillin concentrations (0, 2, 10, 50, 250, and 1000 µm, MB4282, Meilunbio, China) in 120 hpf and at different stages of embryo. The concentration gradients were set around IC50 based on literature reports and median lethal concentration (LC_50_). All pharmaceuticals were initially dissolved in dimethyl sulfoxide (DMSO) and subsequently diluted in E3 medium to achieve the desired final concentrations (DMSO<0.1%). The control group was exposed to E3 medium containing 0.1% DMSO. Each treatment group consisted of six biological replicates, with each replicate comprising a 2 L glass beaker containing 1.5 L of exposure solution and 100 embryos.

### 5.2 Mice Husbandry and Amoxicillin Treatment

All animal experiments in this study were approved by the Institutional Animal Care and Use Committee of Zhongnan Hospital of Wuhan University (IACUC No. ZN2022024). All procedures were conducted in accordance with the guidelines for the care and use of laboratory animals issued by the Chinese Laboratory Animal Welfare and Ethics Committee. Specific pathogen‐free (SPF) male and female Kunming mice were acclimated for 7 days under standardized housing conditions, with the temperature maintained at 18°C–22°C, relative humidity at 40%–60%, and a 12 h light/12 h dark cycle. Subsequently, female and male mice were housed together at a ratio of 2:1 at 17:00 each day. Vaginal smears were collected the following morning and examined microscopically, and the day on which sperm were detected was designated as gestational day 0. Pregnant mice were randomly assigned to four groups: CON, Am, VbL, and VbH groups, with eight dams in each group.

To simulate the timing of clinical amoxicillin use during pregnancy, pregnant mice were administered amoxicillin and/or vitamin B1 from GD10 to GD18. In the Am group, pregnant mice received amoxicillin solution by oral gavage at 300 mg kg^−1^ day^−1^ once daily from GD10 to GD18, with the maximum gavage volume maintained below 0.4 mL per 10 g body weight. In the ABL group, pregnant mice received amoxicillin at 300 mg kg^−1^ day^−1^ together with vitamin B1 at 0.1 mg kg^−1^ day^−1^ by oral gavage from GD10 to GD18. In the ABH group, pregnant mice received amoxicillin at 300 mg kg^−1^ day^−1^ together with vitamin B1 at 0.2 mg kg^−1^ day^−1^ by oral gavage from GD10 to GD18. The CON group received an equivalent volume of normal saline. The maximum gavage volume in all treatment groups was maintained below 0.4 mL per 10 g body weight.

On GD19, pregnant mice were anesthetized with 2.5% isoflurane and euthanized. Maternal and fetal blood samples, adrenal glands, hearts, livers, and brain tissues were collected. Pregnancy outcomes were recorded, and fetal body length, tail length, body weight, and placental weight were measured. From each litter, three fetuses were randomly selected for tissue fixation, and each organ was fixed in 4% paraformaldehyde. The remaining samples were snap‐frozen in liquid nitrogen and stored at −80°C for subsequent analyses.

### 5.3 Morphological Data Measurements

Before bright‐field imaging, 72, 96, 120 h post‐fertilization (hpf) zebrafish larvae were placed in a 90 mm Petri dish (n = 3 or 6 per group), water was added to the fish body, 1 × tricaine was added, the dish was tapped until the larvae showed no obvious activity, and then observed under a type microscope. Images were taken with an Olympus AH‐2 optical microscope (Tokyo, Japan). Phenotypes were quantified with the 1.53c ImageJ software using body length, head length, head area, and eye area. The experiment was conducted in triplicate.

### 5.4 Quantitative Real‐Time PCR

Total RNA was extracted from whole zebrafish larvae and separated mouse tissues using the TRIzol method (15596026CN, Thermo Fisher Scientific, USA). Reverse transcription was performed using a reverse transcription kit (R323‐01, Vazyme, China), followed by quantitative real‐time polymerase chain reaction (qPCR) using the SYBR Green qPCR Master Mix Kit (Q712‐02, Vazyme, China). Organ‐specific functional markers were detected in the total RNA of the whole larvae. The results were calculated using the 2^−ΔΔCt^ method, with normalization to the cox4i gene. The corresponding primers are listed in Table S1.

### 5.5 The Light/Dark Test

Zebrafish larvae were acclimated for 30 min in a 24‐well plate, with one fish per well. After acclimation, individual fish were tracked in the wells using Noldus software (Noldus, Netherlands). The movement trajectories of each larva were recorded for 60 min, with three light/dark cycles (each cycle consisting of 10 min of light followed by 10 min of darkness). The behavioral changes of zebrafish larvae were assessed by calculating the activity distance and the total duration of active time during the light phase.

### 5.6 In Situ Hybridization

In situ hybridization was performed using antisense RNA probes following standard protocols. cDNA fragments were extracted from 120 hpf larvae as templates, and probe primers were designed to amplify a probe targeting *nr5a1a* expression. The partial coding sequence of *nr5a1a* was amplified using the forward primer 5′‐AGTGTCCCCATAAACCCTGC‐3′ and the reverse primer 5′‐GGTAATACGACTCACTATAGG CCGCTTCACCCTACTGGTTA‐3′. The probe template was amplified using 2× Easy Tag PCR Supermix (AS111‐01, TransGen Biotech, China) and purified with the QIAquick PCR Purification Kit (28104, QIAGEN, Germany). DIG‐labeled RNA probes were synthesized via in vitro transcription using a DIG RNA Labeling Kit (11277073910, Roche, Switzerland). For hybridization, fixed embryos were incubated with RNA probes overnight at 4°C. Subsequently, an antidigoxigenin alkaline phosphatase‐conjugated antibody (11093274910, Roche, Switzerland) was added to bind the DIG‐labeled RNA probes. Finally, NBT/BCIP solution (11681451001, Roche, Switzerland) was used to induce a colorimetric reaction, revealing color changes associated with the target gene's expression. After hybridization, embryo images were captured using an Olympus optical microscope.

### 5.7 Plasmid Construction and Microinjection

The full‐length PCALRx sequence was chemically synthesized and cloned into a Tol2‐based over‐expression vector containing an independent EGFP reporter cassette. The PCALRx and EGFP expression cassettes were driven by separate promoters and terminated by independent polyadenylation signals; therefore, PCALRx and EGFP were transcribed as separate RNA molecules rather than as a fusion transcript. The PCALRx over‐expression plasmid was co‐injected with 30 pg of Tol2 transposase mRNA into one‐cell‐stage embryos to induce transient PCALRx over‐expression in EGFP‐positive zebrafish embryos. The PCALRx over‐expression plasmid and the corresponding negative‐control plasmid (NC; an otherwise identical empty vector containing the independent EGFP reporter cassette but lacking the PCALRx insert; Gene Tools, USA) were microinjected into zebrafish embryos at the one‐cell stage using glass microinjection needles (inner tip diameter 0.07 mm). The injection volume was 1.5–2 nL per embryo, and the sham‐operated group was injected with the same volume of sterile water to exclude non‐specific effects caused by the microinjection procedure itself, including needle penetration, injection volume, embryonic envelope damage, or altered chorion permeability. Zebrafish larvae were collected at 120 hpf for further analysis. Plasmid sequence information is provided in Table S2. The zebrafish PC‐MO was provided by the Gene Tools Company. The PC‐MO and NC powders were diluted with ddH_2_O according to the instructions to prepare the working solution for microinjection.

### 5.8 Transmission Electron Microscopy (TEM)

Fresh 120 hpf zebrafish larvae were fixed in 2.5% glutaraldehyde, post‐fixed in 1% OsO_4_, and dehydrated with gradient ethanol and acetone. The tissues were embedded in epoxy resin, cut into serial TEM ultrathin sections (approximately 70 nm thick), and stained with uranyl acetate and lead citrate. The sections were photographed using a JEM 1400 JEOL instrument (Tokyo, Japan).

### 5.9 RNA–Protein Pull‐Down Assay

RNA–protein interactions were analyzed using the Pierce Magnetic RNA‐Protein Pull‐Down Kit (Thermo Scientific) according to the manufacturer's instructions with minor modifications. Briefly, target RNA was 3′ end‐labeled with desthiobiotin and purified prior to use. Desthiobiotinylated RNA (50 pmol) was immobilized on streptavidin magnetic beads and incubated with cell lysates (20–200 µg total protein) at 4°C to allow RNA–protein complex formation. Control group used a negative RNA control [poly(A)25 RNA] as an RNA probe. After extensive washing to remove nonspecific binding, RNA‐associated proteins were eluted using biotin elution buffer. Eluted proteins were subsequently analyzed by Western blotting or mass spectrometry as indicated. Control pull‐downs were performed using beads alone or labeled control RNA.

### 5.10 Western Blot Analysis

For Western blot analysis, 50 zebrafish larvae at 120 hpf were collected for each sample. Samples were lysed in protein lysis buffer containing 1% protease inhibitor at a tissue‐to‐lysis buffer ratio of 1:10 and homogenized thoroughly using a tissue homogenizer. After incubation on ice for 20–30 min, the lysates were centrifuged at 12 000 rpm for 5 min at 4°C. The supernatants were collected into pre‐chilled tubes, and protein concentrations were determined using the BCA assay. After normalization of protein concentrations, samples were mixed with 5× SDS‐PAGE loading buffer and denatured at 100°C for 10 min, followed by storage at −20°C until use.

Equal amounts of protein sample were separated by 10% SDS‐PAGE and transferred onto PVDF membranes using wet transfer. The membranes were blocked with 5% non‐fat milk at room temperature for 2 h and then incubated overnight at 4°C with primary antibodies against PC and β‐actin. After three washes with PBST, the membranes were incubated with horseradish peroxidase‐conjugated secondary antibodies at room temperature for 2 h. Protein bands were visualized using an ECL chemiluminescence detection system. Band intensities were quantified using Image‐Pro Plus 6.0 and normalized to β‐actin.

### 5.11 RNA Fluorescence In Situ Hybridization (RNA‐FISH)

RNA‐FISH was performed to detect the expression in zebrafish larvae. Briefly, samples were fixed with 4% paraformaldehyde (PFA) at 4°C for 2–4 h, washed with PBS, dehydrated in 30% sucrose solution, embedded in paraffin, and sectioned at 4–6 µm thickness. Paraffin sections were incubated at 60°C for 1 h, followed by deparaffinization and rehydration using xylene and graded ethanol solutions. Sections were refixed with 4% PFA for 10 min, permeabilized with 0.2% Triton X‐100 for 10 min, and treated with Proteinase K (1–5 µg mL^−1^) for 5–10 min to enhance probe accessibility. After refixation, sections were prehybridized with hybridization buffer at 37°C for 30–60 min. Green fluorescently labeled antisense RNA probes specifically targeting PCALRx transcripts were diluted in hybridization buffer (0.5–1.0 µm) and applied to sections for overnight hybridization at 37°C in a humidified chamber protected from light. After hybridization, sections were washed sequentially with 2× SSC containing 50% formamide, followed by 2× SSC, 0.5× SSC, and 0.1× SSC at 37°C under light‐protected conditions. Nuclei were counterstained with DAPI (1 µg mL^−1^), and sections were mounted using antifade mounting medium. Fluorescence images were acquired using a confocal microscope with identical acquisition parameters for all groups. Images were processed and quantitatively analyzed using consistent settings.

### 5.12 Co‐immunoprecipitation Analysis of PC Ubiquitination

For co‐immunoprecipitation analysis of PC ubiquitination, ZF4 cells were cultured under the indicated treatment conditions and treated with MG132 before collection to preserve ubiquitinated proteins. Cells were washed with cold PBS and lysed on ice in IP lysis buffer containing protease inhibitors and N‐ethylmaleimide. After centrifugation at 12 000 g for 10 min at 4°C, the supernatants were collected, and protein concentrations were determined using the BCA assay. Equal amounts of total protein were incubated overnight at 4°C with anti‐PC antibody or normal IgG as a negative control, followed by incubation with protein A/G magnetic beads. The immune complexes were washed several times with cold lysis buffer and eluted by boiling in SDS‐PAGE loading buffer. The eluted proteins were separated by SDS‐PAGE and transferred onto PVDF membranes. Ubiquitinated PC was detected by immunoblotting with an anti‐ubiquitin antibody, while PC was detected as the immunoprecipitation control. Input lysates were analyzed in parallel to assess total PC and β‐actin levels.

### 5.13 Cycloheximide Chase Assay

For the cycloheximide chase assay, ZF4 cells were treated under the indicated experimental conditions and then incubated with cycloheximide (CHX, 50 µg mL^−1^) to inhibit new protein synthesis. Cells were collected at 0, 2, 4, and 8 h after CHX treatment and lysed in protein extraction buffer containing protease inhibitors. Protein concentrations were determined using the BCA assay, and equal amounts of protein were subjected to SDS‐PAGE and Western blotting. PC protein levels were detected using an anti‐PC antibody, with β‐actin used as the loading control. The relative PC protein abundance at each time point was normalized to the 0 h level to evaluate the degradation rate of PC protein.

### 5.14 Drug Repurposing Screening

A drug repurposing strategy was applied to identify compounds capable of alleviating amoxicillin‐induced metabolic disturbances. Candidate compounds were selected from a library of FDA‐approved or well‐characterized drugs with known metabolic regulatory activity, leveraging existing safety and pharmacokinetic information to accelerate screening. Initial screening was performed using a phenotypic, cell‐based assay. ZF4 cells were exposed to amoxicillin and subsequently treated with individual candidate compounds, followed by quantification of key metabolic endpoints using targeted metabolite analysis. Compounds that significantly mitigated amoxicillin‐induced metabolic alterations were prioritized for further evaluation. Selected hits were subsequently validated in the zebrafish embryonic exposure model by assessing developmental and organ‐specific endpoints. This phenotypic screening approach enabled identification of compounds that modulate complex metabolic responses without prior assumptions regarding molecular targets.

### 5.15 Immunofluorescence Staining

After deparaffinizing zebrafish larvae tissue sections, the slides were treated with 0.3% Triton X‐100 for antigen retrieval. Sections were then washed with PBS and blocked with 10% goat serum (AR1009, Boster Biological Technology Co., Ltd., China) at room temperature for 2 h. The sections were incubated overnight at 4°C with pyruvate carboxylase (PC, dilution 1:100, A6301, Abclonal, China) primary antibodies. After three PBS washes, the sections were incubated with fluorescent secondary antibodies, followed by DAPI staining and application of fluorescence anti‐fade reagent. Images were taken using a Leica laser scanning confocal microscope (TCS SP8, Germany), and image merging and quantitative analysis were performed using ImageJ software.

### 5.16 Statistical Analysis

All quantitative data were statistically analyzed using GraphPad Prism 9.0 and are presented as mean ± S.E.M. A two‐tailed unpaired *t*‐test was used for comparison between two independent groups. For data sets with more than two independent samples, one‐way analysis of variance (ANOVA) was performed, followed by Tukey's multiple comparison test. *P* values were calculated based on the type of data, and differences were considered statistically significant when *P*<0.05.

## Author Contributions

**Yixue Yao** completed most of the experiments and wrote the first revision of article. **Ping Jiang** provided help with revising the manuscript and completed the supplementary experiment. **Xinli Zhou** provided help with zebrafish behavior test and microinjection. **Mengying Yin** and **Qiong Luo** provided help with bred animals and sacrificed. **Hui Wang** guided all experiments and revised the manuscript. All authors have given approval to the final version of the manuscript.

## Funding

The National Key Research and Development Program of China (No. 2020YFA0803900).

## Supporting information




**Supporting File**: advs76937‐sup‐0001‐SuppMat.docx.

## Data Availability

The data that support the findings of this study are available from the corresponding author upon reasonable request.

## References

[1] M. Bilal , M. Adeel , T. Rasheed , Y. Zhao , and H. M. N. Iqbal , “Emerging Contaminants of High Concern and Their Enzyme‐Assisted Biodegradation—A Review,” Environment International 124 (2019): 336–353, 10.1016/j.envint.2019.01.011. 30660847

[2] X. Liu , S. Lu , W. Guo , B. Xi , and W. Wang , “Antibiotics in the Aquatic Environments: A Review of Lakes, China,” Science of The Total Environment 627 (2018): 1195–1208, 10.1016/j.scitotenv.2018.01.271. 30857084

[3] T. P. Van Boeckel , C. Brower , M. Gilbert , et al., “Global Trends in Antimicrobial Use in Food Animals,” PNAS 112, no. 18 (2015): 5649–5654, 10.1073/pnas.1503141112. 25792457 PMC4426470

[4] J. P. Bavumiragira , J. Ge , and H. Yin , “Fate and Transport of Pharmaceuticals in Water Systems: A Processes Review,” Science of The Total Environment 823 (2022): 153635, 10.1016/j.scitotenv.2022.153635. 35124044

[5] M. Taheran , M. Naghdi , S. K. Brar , M. Verma , and R. Y. Surampalli , “Emerging Contaminants: Here Today, There Tomorrow! Environmental Nanotechnology,” Monitoring & Management 10 (2018): 122–126, 10.1016/j.enmm.2018.05.010.

[6] X. Zeng , L. Zhang , Q. Chen , et al., “Maternal Antibiotic Concentrations in Pregnant Women in Shanghai and Their Determinants: A Biomonitoring‐Based Prospective Study,” Environment International 138 (2020): 105638, 10.1016/j.envint.2020.105638. 32179319

[7] Q. Q. Zhang , G. G. Ying , C. G. Pan , Y. S. Liu , and J. L. Zhao , “Comprehensive Evaluation of Antibiotics Emission and Fate in the River Basins of China: Source Analysis, Multimedia Modeling, and Linkage to Bacterial Resistance,” Environmental Science & Technology 49, no. 11 (2015): 6772–6782, 10.1021/acs.est.5b00729. 25961663

[8] L. J. Zhou , G. G. Ying , S. Liu , et al., “Occurrence and Fate of Eleven Classes of Antibiotics in Two Typical Wastewater Treatment Plants in South China,” Science of The Total Environment 452‐453 (2013): 365–376, 10.1016/j.scitotenv.2013.03.010. 23538107

[9] H. Wang , L. Ren , X. Yu , et al., “Antibiotic Residues in Meat, Milk and Aquatic Products in Shanghai and human Exposure Assessment,” Food Control 80 (2017): 217–225, 10.1016/j.foodcont.2017.04.034.

[10] M. Bacanlı and N. Başaran , “Importance of Antibiotic Residues in Animal Food,” Food and Chemical Toxicology 125 (2019): 462–466, 10.1016/j.fct.2019.01.033. 30710599

[11] S. Rodriguez‐Mozaz , S. Chamorro , E. Marti , et al., “Occurrence of Antibiotics and Antibiotic Resistance Genes in Hospital and Urban Wastewaters and Their Impact on the Receiving River,” Water Research 69 (2015): 234–242, 10.1016/j.watres.2014.11.021. 25482914

[12] H. Rac , A. P. Gould , L. S. Eiland , et al., “Common Bacterial and Viral Infections: Review of Management in the Pregnant Patient,” Annals of Pharmacotherapy 53, no. 6 (2019): 639–651, 10.1177/1060028018817935. 30556401

[13] J. E. Allsworth and J. F. Peipert , “Prevalence of Bacterial Vaginosis,” Obstetrics & Gynecology 109, no. 1 (2007): 114–120, 10.1097/01.Aog.0000247627.84791.91. 17197596

[14] A. Huttner , J. Bielicki , M. N. Clements , et al., “Oral Amoxicillin and Amoxicillin–clavulanic Acid: Properties, Indications and Usage,” Clinical Microbiology and Infection 26, no. 7 (2020): 871–879, 10.1016/j.cmi.2019.11.028. 31811919

[15] A. Arancibia , J. Guttmann , G. González , and C. González , “Absorption and Disposition Kinetics of Amoxicillin in Normal human Subjects,” Antimicrobial Agents and Chemotherapy 17, no. 2 (1980): 199–202, 10.1128/aac.17.2.199. 7387142 PMC283758

[16] A. K. Thabit , D. F. Fatani , M. S. Bamakhrama , O. A. Barnawi , L. O. Basudan , and S. F. Alhejaili , “Antibiotic Penetration into Bone and Joints: An Updated Review,” International Journal of Infectious Diseases 81 (2019): 128–136, 10.1016/j.ijid.2019.02.005. 30772469

[17] J. Schnarr and F. Smaill , “Asymptomatic Bacteriuria and Symptomatic Urinary Tract Infections in Pregnancy,” European Journal of Clinical Investigation 38, no. s2 (2008): 50–57, 10.1111/j.1365-2362.2008.02009.x. 18826482

[18] J. Lenzer , “Centers for Disease Control and Prevention: Protecting the Private Good?,” BMJ 350 (2015): h2362, 10.1136/bmj.h2362. 25979454

[19] Y. Ansaldi and B. M. de Tejada Weber , “Urinary Tract Infections in Pregnancy,” Clinical Microbiology and Infection 29, no. 10 (2023): 1249–1253, 10.1016/j.cmi.2022.08.015. 36031053

[20] A. Rau , J. Keri , and J. E. Murase , “Management of Acne in Pregnancy,” American Journal of Clinical Dermatology 25, no. 3 (2024): 465–471, 10.1007/s40257-024-00851-6. 38453786

[21] R. E. Bray , R. W. Boe , and W. L. Johnson , “Transfer of Ampicillin into Fetus and Amniotic Fluid from Maternal Plasma in Late Pregnancy,” American Journal of Obstetrics and Gynecology 96, no. 7 (1966): 938–942, 10.1016/0002-9378(66)90438-8. 5928457

[22] M. A. MacAulay , M. Abou‐Sabe , and D. Charles , “Placental Transfer of Ampicillin,” American Journal of Obstetrics and Gynecology 96, no. 7 (1966): 943–950, 10.1016/0002-9378(66)90439-x. 5928458

[23] A. E. Czeizel , M. Rockenbauer , H. T. Sørensen , and J. Olsen , “Augmentin Treatment during Pregnancy and the Prevalence of Congenital Abnormalities,” European Journal of Obstetrics & Gynecology and Reproductive Biology 97, no. 2 (2001): 188–192, 10.1016/s0301-2115(00)00545-5. 11451547

[24] M. Reyman , M. A. van Houten , R. L. Watson , et al., “Effects of Early‐Life Antibiotics on the Developing Infant Gut Microbiome and Resistome: A Randomized Trial,” Nature Communications 13, no. 1 (2022): 893, 10.1038/s41467-022-28525-z. PMC8850541 35173154

[25] M. F. Endika , D. J. M. Barnett , C. E. Klostermann , et al., “Microbiota‐Dependent Influence of Prebiotics on the Resilience of Infant Gut Microbiota to Amoxicillin/Clavulanate Perturbation in an in Vitro Colon Model,” Frontiers in Microbiology 14 (2023): 1131953, 10.3389/fmicb.2023.1131953. 37275167 PMC10232780

[26] J. Zhao , K. Yao , H. Yu , et al., “Metabolic Remodelling during Early Mouse Embryo Development,” Nature Metabolism 3, no. 10 (2021): 1372–1384, 10.1038/s42255-021-00464-x. 34650276

[27] V. Jain , V. Prakash , G. Sagar , et al., “Metabolomics Approach to Evaluate Diclazuril‐Induced Developmental Toxicity in Zebrafish Embryo,” Aquatic Toxicology 279 (2025): 107238, 10.1016/j.aquatox.2025.107238. 39813883

[28] Z. Zhang , H. Shi , K. Zhang , et al., “Transcriptome‐Guided Characterization of the Environmental Toxicity of Metformin: Disruption of Energy Homeostasis and Inhibition of Embryonic Development of Zebrafish at Environmentally Relevant Concentrations,” Environmental Science & Technology 58, no. 40 (2024): 17580–17591, 10.1021/acs.est.4c05052. 39319773

[29] W. Li , B. Xia , X. Geng , X. Liu , and M. Wang , “Prenatal Amoxicillin Exposure Impairs Mouse Alveolar Development via Tet3‐Mediated Hypo‐Hydroxymethylation of Nkx2‐1 Promoter,” Toxicology and Applied Pharmacology 511 (2026): 117817, 10.1016/j.taap.2026.117817. 41967612

[30] S. Lalanne , G. Bouzillé , C. Tron , et al., “Amoxicillin‐Induced Neurotoxicity: Contribution of a Healthcare Data Warehouse to the Determination of a Toxic Concentration Threshold,” Antibiotics (Basel) 12, no. 4 (2023): 680, 10.3390/antibiotics12040680. 37107042 PMC10135267

[31] P. Gao , C. Chang , J. Liang , F. Du , and R. Zhang , “Embryonic Amoxicillin Exposure Has Limited Impact on Liver Development but Increases Susceptibility to NAFLD in Zebrafish Larvae,” International Journal of Molecular Sciences 25, no. 5 (2024): 2744, 10.3390/ijms25052744. 38473993 PMC10931932

[32] A. B. Herman , D. Tsitsipatis , and M. Gorospe , “Integrated lncRNA Function Upon Genomic and Epigenomic Regulation,” Molecular Cell 82, no. 12 (2022): 2252–2266, 10.1016/j.molcel.2022.05.027. 35714586 PMC9219586

[33] M. Wachsmuth , M. Caudron‐Herger , and K. Rippe , “Genome Organization: Balancing Stability and Plasticity,” Biochimica et Biophysica Acta (BBA)—Molecular Cell Research 1783, no. 11 (2008): 2061–2079, 10.1016/j.bbamcr.2008.07.022. 18722483

[34] Q. Zhao , J. Liu , H. Deng , et al., “Targeting Mitochondria‐Located circRNA SCAR Alleviates NASH via Reducing mROS Output,” Cell 183, no. 1 (2020): 76–93.e22, 10.1016/j.cell.2020.08.009. 32931733

[35] K. V. Tran , E. L. Brown , T. DeSouza , et al., “Human Thermogenic Adipocyte Regulation by the Long Noncoding RNA LINC00473,” Nature Metabolism 2, no. 5 (2020): 397–412, 10.1038/s42255-020-0205-x. PMC7241442 32440655

[36] A. G. Kerr , Z. Wang , N. Wang , et al., “The Long Noncoding RNA ADIPINT Regulates human Adipocyte Metabolism via Pyruvate Carboxylase,” Nature Communications 13, no. 1 (2022): 2958, 10.1038/s41467-022-30620-0. PMC9135762 35618718

[37] A. Aqel , A. Alnesf , I. I. Aigha , et al., “Glucokinase (GCK) in Diabetes: From Molecular Mechanisms to Disease Pathogenesis,” Cellular & Molecular Biology Letters 29, no. 1 (2024): 120, 10.1186/s11658-024-00640-3. 39245718 PMC11382428

[38] S. Pushpakom , F. Iorio , P. A. Eyers , et al., “Drug Repurposing: Progress, Challenges and Recommendations,” Nature Reviews Drug Discovery 18, no. 1 (2019): 41–58, 10.1038/nrd.2018.168. 30310233

[39] M. A. Farha and E. D. Brown , “Drug Repurposing for Antimicrobial Discovery,” Nature Microbiology 4, no. 4 (2019): 565–577, 10.1038/s41564-019-0357-1. 30833727

[40] C. Chu , Q. C. Zhang , S. T. da Rocha , et al., “Systematic Discovery of Xist RNA Binding Proteins,” Cell 161, no. 2 (2015): 404–416, 10.1016/j.cell.2015.03.025. 25843628 PMC4425988

[41] L. Liu , Y. Hong , C. Ma , et al., “Circular RNA Gtdc1 Protects against Offspring Osteoarthritis Induced by Prenatal Prednisone Exposure by Regulating SRSF1‐Fn1 Signaling,” Advanced Science 11, no. 20 (2024): 2307442, 10.1002/advs.202307442. 38520084 PMC11132075

[42] Y. Chen , H. Qu , X. Li , and H. Wang , “Effects of Amoxicillin Exposure at Different Stages, Doses and Courses of Pregnancy on Adrenal Development in Fetal Mice,” Food and Chemical Toxicology 175 (2023): 113754, 10.1016/j.fct.2023.113754. 37001632

[43] Y. Dai , Y. Peng , W. Hu , Y. Liu , and H. Wang , “Prenatal Amoxicillin Exposure Induces Developmental Toxicity in Fetal Mice and Its Characteristics,” Journal of Environmental Sciences 137 (2024): 287–301, 10.1016/j.jes.2023.02.021. 37980015

[44] H. He , M. Chen , F. Long , et al., “The “Toxic Window” of Amoxicillin Exposure during Pregnancy on Long Bone Development in Fetal Mice,” Life Sciences 350 (2024): 122759, 10.1016/j.lfs.2024.122759. 38815897

[45] P. J. Hotez , M. E. Bottazzi , N. Y. Islam , et al., “The Zebrafish as a Potential Model for Vaccine and Adjuvant Development,” Expert Review of Vaccines 23, no. 1 (2024): 535–545, 10.1080/14760584.2024.2345685. 38664959

[46] W. Dong , I. Akasaka , A. Komiyama , et al., “Augmentation of Pectoral Fin Teratogenicity by Thalidomide in Human Cytochrome P450 3A‐Expressing Zebrafish,” Pharmaceuticals 16, no. 3 (2023): 368, 10.3390/ph16030368. 36986467 PMC10055635

[47] K. Howe , M. D. Clark , C. F. Torroja , et al., “The Zebrafish Reference Genome Sequence and Its Relationship to the Human Genome,” Nature 496, no. 7446 (2013): 498–503, 10.1038/nature12111. 23594743 PMC3703927

[48] F. Shihana , P. M. Cholan , S. Fraser , S. H. Oehlers , and D. Seth , “Investigating the Role of Lipid Genes in Liver Disease Using Fatty Liver Models of Alcohol and High Fat in Zebrafish (Danio rerio),” Liver International 43, no. 11 (2023): 2455–2468, 10.1111/liv.15716. 37650211

[49] G. R. Garcia , P. D. Noyes , and R. L. Tanguay , “Advancements in Zebrafish Applications for 21st Century Toxicology,” Pharmacology & Therapeutics 161 (2016): 11–21, 10.1016/j.pharmthera.2016.03.009. 27016469 PMC4851906

[50] J. H. He , J. M. Gao , C. J. Huang , and C. Q. Li , “Zebrafish Models for Assessing Developmental and Reproductive Toxicity,” Neurotoxicology and Teratology 42 (2014): 35–42, 10.1016/j.ntt.2014.01.006. 24503215

[51] F. Mahmood , S. Fu , J. Cooke , S. W. Wilson , J. D. Cooper , and C. Russell , “A Zebrafish Model of CLN2 Disease Is Deficient in Tripeptidyl Peptidase 1 and Displays Progressive Neurodegeneration Accompanied by a Reduction in Proliferation,” Brain 136, no. 5 (2013): 1488–1507, 10.1093/brain/awt043. 23587805

[52] D. Staudt and D. Stainier , “Uncovering the Molecular and Cellular Mechanisms of Heart Development Using the Zebrafish,” Annual Review of Genetics 46, no. 1 (2012): 397–418, 10.1146/annurev-genet-110711-155646. PMC6982417 22974299

[53] J. Zareba‐Szczudlik , E. Romejko‐Wolniewicz , Z. Lewandowski , et al., “Evaluation of the Amoxicillin Concentrations in Amniotic Fluid, Placenta, Umbilical Cord Blood and Maternal Serum Two Hours after Oral Administration,” Neuro Endocrinology Letters 38, no. 7 (2017): 502–508. 29369602

[54] A. Usai , G. Di Franco , P. Colucci , et al., “A Model of a Zebrafish Avatar for Co‐Clinical Trials,” Cancers (Basel) 12, no. 3 (2020): 677, 10.3390/cancers12030677. 32183229 PMC7140063

[55] R. Oliveira , S. McDonough , J. C. Ladewig , A. M. Soares , A. J. Nogueira , and I. Domingues , “Effects of Oxytetracycline and Amoxicillin on Development and Biomarkers Activities of Zebrafish (Danio rerio),” Environmental Toxicology and Pharmacology 36, no. 3 (2013): 903–912, 10.1016/j.etap.2013.07.019. 24008007

[56] D. Raldúa and B. Piña , “In Vivo Zebrafish Assays for Analyzing Drug Toxicity,” Expert Opinion on Drug Metabolism & Toxicology 10, no. 5 (2014): 685–697, 10.1517/17425255.2014.896339. 24617455

[57] C.‐L. Zhang , Y. Zou , W. He , F. H. Gage , and R. M. Evans , “A Role for Adult TLX‐Positive Neural Stem Cells in Learning and Behavior,” Nature 451, no. 7181 (2008): 1004–1007, 10.1038/nature06562. 18235445

[58] I. Bacila , V. T. Cunliffe , and N. P. Krone , “Interrenal Development and Function in Zebrafish,” Molecular and Cellular Endocrinology 535 (2021): 111372, 10.1016/j.mce.2021.111372. 34175410

[59] J. Chu and K. C. Sadler , “New School in Liver Development: Lessons from zebrafish #,” Hepatology 50, no. 5 (2009): 1656–1663, 10.1002/hep.23157. 19693947 PMC3093159

[60] B. R. Keegan , J. L. Feldman , G. Begemann , P. W. Ingham , and D. Yelon , “Retinoic Acid Signaling Restricts the Cardiac Progenitor Pool,” Science 307, no. 5707 (2005): 247–249, 10.1126/science.1101573. 15653502

[61] D. J. Barker , “The Origins of the Developmental Origins Theory,” Journal of Internal Medicine 261, no. 5 (2007): 412–417, 10.1111/j.1365-2796.2007.01809.x. 17444880

[62] N. Delgado , J. Orozco , S. Zambrano , J. C. Casas‐Zapata , and D. Marino , “Veterinary Pharmaceutical as Emerging Contaminants in Wastewater and Surface Water: An Overview,” Journal of Hazardous Materials 460 (2023): 132431, 10.1016/j.jhazmat.2023.132431. 37688873

[63] A. J. Watkinson , E. J. Murby , D. W. Kolpin , and S. D. Costanzo , “The Occurrence of Antibiotics in an Urban Watershed: From Wastewater to Drinking Water,” Science of The Total Environment 407, no. 8 (2009): 2711–2723, 10.1016/j.scitotenv.2008.11.059. 19138787

[64] M. A. Locatelli , F. F. Sodré , and W. F. Jardim , “Determination of Antibiotics in Brazilian Surface Waters Using Liquid Chromatography–Electrospray Tandem Mass Spectrometry,” Archives of Environmental Contamination and Toxicology 60, no. 3 (2011): 385–393, 10.1007/s00244-010-9550-1. 20535610

[65] K. Balakrishna , A. Rath , Y. Praveenkumarreddy , K. S. Guruge , and B. Subedi , “A Review of the Occurrence of Pharmaceuticals and Personal Care Products in Indian Water Bodies,” Ecotoxicology and Environmental Safety 137 (2017): 113–120, 10.1016/j.ecoenv.2016.11.014. 27915141

[66] C. Wang , D. Ye , X. Li , et al., “Occurrence of Pharmaceuticals and Personal Care Products in Bottled Water and Assessment of the Associated Risks,” Environment International 155 (2021): 106651, 10.1016/j.envint.2021.106651. 34033976

[67] N. H. Tran , L. Hoang , L. D. Nghiem , et al., “Occurrence and Risk Assessment of Multiple Classes of Antibiotics in Urban Canals and Lakes in Hanoi, Vietnam,” Science of The Total Environment 692 (2019): 157–174, 10.1016/j.scitotenv.2019.07.092. 31344569

[68] D. C. Wallace , “A Mitochondrial Bioenergetic Etiology of Disease,” Journal of Clinical Investigation 123, no. 4 (2013): 1405–1412, 10.1172/jci61398. 23543062 PMC3614529

[69] P. Monnier , C. Martinet , J. Pontis , I. Stancheva , S. Ait‐Si‐Ali , and L. Dandolo , “H19 lncRNA Controls Gene Expression of the Imprinted Gene Network by Recruiting MBD1,” Proceedings of the National Academy of Sciences 110, no. 51 (2013): 20693–20698, 10.1073/pnas.1310201110. PMC3870736 24297921

[70] H. Sunwoo , M. E. Dinger , J. E. Wilusz , P. P. Amaral , J. S. Mattick , and D. L. Spector , “MEN ε/β Nuclear‐Retained Non‐Coding RNAs Are Up‐Regulated Upon Muscle Differentiation and Are Essential Components of Paraspeckles,” Genome Research 19, no. 3 (2009): 347–359, 10.1101/gr.087775.108. 19106332 PMC2661813

[71] F. Bantignies , V. Roure , I. Comet , et al., “Polycomb‐Dependent Regulatory Contacts between Distant Hox Loci in Drosophila,” Cell 144, no. 2 (2011): 214–226, 10.1016/j.cell.2010.12.026. 21241892

[72] Z. Lv , Z. Wang , J. Hu , et al., “LncRNA PVT1 Induces Mitochondrial Dysfunction of Podocytes via TRIM56 in Diabetic Kidney Disease,” Cell Death & Disease 15, no. 9 (2024): 697, 10.1038/s41419-024-07107-5. 39349450 PMC11442824

[73] S. Jitrapakdee , A. Vidal‐Puig , and J. C. Wallace , “Anaplerotic Roles of Pyruvate Carboxylase in Mammalian Tissues,” Cellular and Molecular Life Sciences 63, no. 7‐8 (2006): 843–854, 10.1007/s00018-005-5410-y. 16505973 PMC11136034

[74] J. Kaźmierczak‐Barańska , K. Halczuk , and B. T. Karwowski , “Thiamine (Vitamin B1)‐An Essential Health Regulator,” Nutrients 17, no. 13 (2025): 2206, 10.3390/nu17132206. 40647310 PMC12251314

[75] M. Serra , R. Mollace , G. Ritorto , et al., “A Systematic Review of Thiamine Supplementation in Improving Diabetes and Its Related Cardiovascular Dysfunction,” International Journal of Molecular Sciences 26, no. 9 (2025): 3932, 10.3390/ijms26093932. 40362174 PMC12072100

[76] J. R. Measelle , D. A. Baldwin , J. Gallant , et al., “Thiamine Supplementation Holds Neurocognitive Benefits for Breastfed Infants during the First Year of Life,” Annals of the New York Academy of Sciences 1498, no. 1 (2021): 116–132, 10.1111/nyas.14610. 34101212 PMC9291201

[77] J. P. Auger , M. Zimmermann , M. Faas , et al., “Metabolic Rewiring Promotes Anti‐Inflammatory Effects of Glucocorticoids,” Nature 629, no. 8010 (2024): 184–192, 10.1038/s41586-024-07282-7. 38600378

[78] R. Wang , M. Halimulati , X. Huang , Y. Ma , L. Li , and Z. Zhang , “Sulforaphane‐Driven Reprogramming of Gut Microbiome and Metabolome Ameliorates the Progression of Hyperuricemia,” Journal of Advanced Research 52 (2023): 19–28, 10.1016/j.jare.2022.11.003. 36371056 PMC10555773

[79] J. N. Meyer , M. C. Leung , J. P. Rooney , et al., “Mitochondria as a Target of Environmental Toxicants,” Toxicological Sciences 134, no. 1 (2013): 1–17, 10.1093/toxsci/kft102. 23629515 PMC3693132

