# Supplementary material for: Long Noncoding RNA PCALRx Interacts with Pyruvate Carboxylase to Drive Multi‐Organ Developmental Toxicity in Zebrafish Embryos Exposed to Amoxicillin
Source: Adv Sci (Weinh). 2026 Aug 5;13(60):e76937. doi: 10.1002/advs.76937 (PMC13440028; doi:10.1002/advs.76937)
Supplement: Supplementary file 1 [file ADVS-13-e76937-s001.docx]

Long Noncoding RNA PCALRx Interacts with Pyruvate Carboxylase to Drive Multi-organ Developmental Toxicity in Zebrafish Embryos Exposed to Amoxicillin

Yixue Yao ^a,#^, Ping Jiang ^a, #^, Xinli Zhou ^a^, Mengying Yin ^a^, Qiong Luo ^a^, Hui Wang ^a,b,*^

^a^ Department of Pharmacology, Wuhan University School of Basic Medical Sciences, Wuhan 430071, China;

^b^ Hubei Provincial Key Laboratory of Developmentally Originated Disease, Wuhan 430071, China
^*^Corresponding author: Hui Wang; E-mail: wanghui19@whu.edu.cn

**In summary：**

**Number of pages, 22; Number of Tables, 2; Number of Figures, 9.**

**1 Supplementary Methods.**

Untargeted Tissue Metabolomic Profiling.

Transcriptomic Sequencing and Data Analysis.

**2 Supplementary Tables.**

Primers and annealing temperature for qPCR.

Sequence of the PCALRx Overexpression Plasmid.

**3 Supplementary Figures.**

Death rate and dose-response morphology of EAE zebrafish larvae.

Effects of low-dose EAE on zebrafish larvae.

Metabolomic and transcriptomic analysis of zebrafish larvae.

Correlation analysis between significantly altered genes and organ-specific functional markers.

Effects of lncRNA PCALRx overexpression and knockdown.

Interaction between lncRNA PCALRx and pyruvate carboxylase

Effects of drug repurposing candidate compounds on ZF4 cell line and zebrafish larvae.

Pregnancy outcomes and organ markers in prenatal amoxicillin exposure offspring of mice.

RNA-FISH staining of mouse organ and effect of PC knockdown in zebrafish larvae.

**1 Supplementary Methods.**

1.1 **Untargeted Tissue Metabolomic Profiling.**

Fresh zebrafish samples were collected at 120 hpf, snap-frozen in liquid nitrogen, and stored at −80°C until analysis. Untargeted metabolomic profiling of frozen zebrafish tissue samples was performed using liquid chromatography–tandem mass spectrometry (LC-MS/MS) by BGI Genomics Co., Ltd. (Wuhan, China). Quality control (QC) samples were prepared by pooling equal volumes from each experimental sample, while blank control samples were prepared by replacing zebrafish tissue homogenates with 53% aqueous methanol and processed using the same procedure as the experimental samples. Briefly, 100 µL of zebrafish tissue homogenate was transferred into an EP tube, mixed with 400 µL of pre-chilled 80% aqueous methanol, vortexed, and incubated on ice for 5 min. The samples were then centrifuged at 15,000 g for 20 min at 4°C. An aliquot of the supernatant was diluted with LC-MS-grade water to adjust the methanol concentration to 53%, followed by centrifugation again under the same conditions. The resulting supernatant was collected for LC-MS/MS analysis. Chromatographic separation was performed on a Hypersil GOLD C18 column at 40°C with a flow rate of 0.2 mL/min. In the positive ion mode, mobile phase A consisted of 0.1% (v/v) formic acid in water, and mobile phase B was methanol. In the negative ion mode, mobile phase A consisted of 5 mM ammonium acetate solution (pH 9.0), and mobile phase B was methanol. The MS scan range was set from m/z 100 to 1500. The electrospray ionization source parameters were as follows: spray voltage, 3.5 kV; sheath gas flow rate, 35 psi; auxiliary gas flow rate, 10 L/min; ion transfer tube temperature, 320°C; S-lens RF level, 60; auxiliary gas heater temperature, 350°C; polarity, positive and negative ion modes. MS/MS data were acquired using data-dependent acquisition.

**1.2 Transcriptomic Sequencing and Data Analysis.**

Whole-body zebrafish samples from the control and embryonic amoxicillin exposure (EAE) groups were collected for transcriptomic sequencing, with each sample containing 200 zebrafish larvae at 120 hpf. RNA sequencing was performed by BGI Genomics Co., Ltd. (Wuhan, China). Total RNA was extracted from whole-body larval tissues, and RNA integrity and quantity were assessed before library preparation. At least 1 μg of total RNA per sample was used for library construction. Poly(A) mRNA was enriched using oligo(dT) magnetic beads and fragmented in fragmentation buffer with divalent cations. First-strand cDNA was synthesized using fragmented mRNA as the template with an M-MuLV reverse transcriptase system, followed by RNA degradation with RNase H and second-strand cDNA synthesis using DNA polymerase I. After purification, double-stranded cDNA was subjected to end repair, A-tailing, adaptor ligation, size selection of 370–420 bp fragments using AMPure XP beads, and PCR amplification to generate the final libraries. Library quality was evaluated using a Qubit 2.0 Fluorometer, Agilent 2100 Bioanalyzer, and RT-qPCR, and libraries with an effective concentration higher than 2 nM were used for sequencing. Qualified libraries were pooled according to their effective concentrations and sequenced on an Illumina platform to generate 150 bp paired-end reads using sequencing-by-synthesis technology. Raw reads were processed by CASAVA base calling and subjected to quality control to remove adaptor-containing reads, reads containing ambiguous nucleotides, and low-quality reads in which bases with Qphred ≤ 20 accounted for more than 50% of the read length. The quality of clean data was assessed based on Q20, Q30, and GC content. Reference genome sequences and gene annotation files were obtained from public genome databases. HISAT2 (v2.0.5) was used to build the genome index and align clean reads to the reference genome. Gene read counts were calculated using FeatureCounts (v1.5.0-p3), and FPKM values were generated according to gene length and read count to estimate gene expression abundance. Differential expression analysis between the control and EAE groups was performed using DESeq2 (v1.20.0), and genes with P < 0.05 and |log2(fold change)| > 1 were considered significantly differentially expressed.

**2 Supplementary Tables.**

**2.1 Table S1. Primers and annealing temperature for qPCR.**

| Gene | Organism | Forward primer | Reverse primer |
| --- | --- | --- | --- |
| *cox4i* | Danio rerio | CACATGGAGTTGCGAAGGTC | TACTAAGCCAGTCAAGCCGAC |
| *gata6* | Danio rerio | GGCACATCGAGGGATACCAG | GAGAGCTGCGGTGTGACATA |
| *foxa3* | Danio rerio | GGGATGTTGAGCTCCGTGAA | GCGCTGGGATAGCCCATATT |
| *hhex* | Danio rerio | CAGCACAGCATTCAGCCTTAAA | ATAAGACGCGATTCTCACCTG |
| *hmgcr* | Danio rerio | TGAGGATGCCGGTTTAAGGG | ATCACTGTGCAATCCTCCGC |
| *fasn* | Danio rerio | AGCAAAGGCCTGTGATGTCC | CGTTTGGACTTTTCGTCTTGCTAT |
| *nr5a1a* | Danio rerio | TTCCCTCCACCAATGCCAAA | GCCCAAACAAAGATGCCAGTC |
| *cyp17a1* | Danio rerio | AGCACTCGTGATGTCGGTTT | TAGATTCCCCCTGTCGCTGA |
| *star* | Danio rerio | GTGCTATGTGCAACAAGGGC | GGTCCATTCTCAGCCCTTACA |
| *gata4* | Danio rerio | GAGCTGCGTCTTACGAGTCC | TGGACACCGGAGAATGATGG |
| *vmhc* | Danio rerio | AACTCTGCCATCTGAGCGTC | GGTTTTTATGCAATTGTGGGCA |
| *amhc* | Danio rerio | ATTCAAGCTAACCCTGCGCT | GCTCGTCCCGAAATGAATGC |
| *sox2* | Danio rerio | TCCATGACCAACTCGCAGAC | GGAATGAGACGACGACGTGA |
| *syn1* | Danio rerio | GAACCTGGCCGGAGATTTTG | ATCACCGGAAAGCTCGTTCA |
| *PCLARx* | Danio rerio | ATGTACTGCTGTAAATCAAG | CAAAAAAAGAGACATACTGC |
| *pcxa* | Danio rerio | TGGAGAGGGCATGGGTATCA | GGCGATGACTTTCACCAGGA |
| *Cox4i* | Mus musculus | ATTGGCAAGAGAGCCATTTCTAC | TGGGGAAAGCATAGTCTTCACT |
| *Gata6* | Mus musculus | TTGCTCCGGTAACAGCAGTG | GTGGTCGCTTGTGTAGAAGGA |
| *Foxa3* | Mus musculus | GCTGACCCTGAGTGAAATCTAC | ACGAAGCAGTCATTGAAGGAC |
| *Hhex* | Mus musculus | GGATGCGTTGGACAGTTTGGA | CTCCTGGTCGGAATCCTCTGA |
| *Hmgcr* | Mus musculus | AGAGCGAGTGCATTAGCAAAG | GATTGCCATTCCACGAGCTAT |
| *Fasn* | Mus musculus | GGAGGTGGTGATAGCCGGTAT | TGGGTAATCCATAGAGCCCAG |
| *Nr5a1a* | Mus musculus | AGGTGTCGGGCTACCACTAC | CCACCCCGCATTCGATCAG |
| *Cyp17a1* | Mus musculus | AGTGCTCGTGAAGAAGGGGA | TTTCCTTGGTCCGACAAGAGG |
| *StAR* | Mus musculus | CGGGTGGATGGGTCAAGTTC | GCACTTCGTCCCCGTTCTC |
| *Gata4* | Mus musculus | CCCTACCCAGCCTACATGG | ACATATCGAGATTGGGGTGTCT |
| *Vmhc* | Mus musculus | ACTGTCAACACTAAGAGGGTCA | TTGGATGATTTGATCTTCCAGGG |
| *Amhc* | Mus musculus | GCCCAGTACCTCCGAAAGTC | GCCTTAACATACTCCTCCTTGTC |
| *Sox2* | Mus musculus | GCGGAGTGGAAACTTTTGTCC | GGGAAGCGTGTACTTATCCTTCT |
| *Syn1* | Mus musculus | CATGGAAGTTCTTCGGAATGGA | GCAAAGAGTTTACACTGGGGAT |

Cox4i, cytochrome c oxidase subunit IV isoform; gata6, GATA binding protein 6; foxa3, forkhead box a3; hhex, hematopoietically expressed homeobox; hmgcr, 3-hydroxy-3-methylglutaryl-coa reductase; fasn, fatty acid synthase; nr5a1a, nuclear receptor subfamily 5, group a, member 1, alpha; cyp17a1, cytochrome p450, family 17, subfamily a, polypeptide 1; star steroidogenic acute regulatory protein; gata4, GATA binding protein 4; vmhc, myosin heavy chain 7; amhc, myh6 myosin, heavy chain 6, cardiac muscle, alpha; sox2, SRY-box transcription factor 2; syn1, synapsin I; pcxa, pyruvate carboxylase alpha.

2.2 Table S2. Sequence of PCALRx Overexpression Plasmid

Sequence Used for Zebrafish PCALRx Overexpression Plasmid Construction

AAGCTTAAACAAGAATCTCTAGTTTTCTTTCTTGCTTTTACTTTTACTTCCTTAATACTCAAGTACAATTTTAATGGAGTACTTTTTTACTTTTACTCAAGTAAGATTCTAGCCAGATACTTTTACTTTTAATTGAGTAAAATTTTCCCTAAGTACTTGTACTTTCACTTGAGTAAAATTTTTGAGTACTTTTTACACCTCTGCTCGACCATATGGGAGAGCTCCCAACGCGTTGGATGCATAGCTTGAGTATTCTATAGTGTCACCTAAATAGCTTGGCGTAATCATGGTCATAGCTGTTTCCTGTGTGAAATTGTTATCCGCTCACAATTCCACACAACATACGAGCCGGAAGCATAAAGTGTAAAGCCTGGGGTGCCTAATGAGTGAGCTAACTCACATTAATTGCGTTGCGCTCACTGCCCGCTTTCCAGTCGGGAAACCTGTCGTGCCAGCTGCATTAATGAATCGGCCAACGCGCGGGGAGAGGCGGTTTGCGTATTGGGCGCTCTTCCGCTTCCTCGCTCACTGACTCGCTGCGCTCGGTCGTTCGGCTGCGGCGAGCGGTATCAGCTCACTCAAAGGCGGTAATACGGTTATCCACAGAATCAGGGGATAACGCAGGAAAGAACATGTGAGCAAAAGGCCAGCAAAAGGCCAGGAACCGTAAAAAGGCCGCGTTGCTGGCGTTTTTCCATAGGCTCCGCCCCCCTGACGAGCATCACAAAAATCGACGCTCAAGTCAGAGGTGGCGAAACCCGACAGGACTATAAAGATACCAGGCGTTTCCCCCTGGAAGCTCCCTCGTGCGCTCTCCTGTTCCGACCCTGCCGCTTACCGGATACCTGTCCGCCTTTCTCCCTTCGGGAAGCGTGGCGCTTTCTCATAGCTCACGCTGTAGGTATCTCAGTTCGGTGTAGGTCGTTCGCTCCAAGCTGGGCTGTGTGCACGAACCCCCCGTTCAGCCCGACCGCTGCGCCTTATCCGGTAACTATCGTCTTGAGTCCAACCCGGTAAGACACGACTTATCGCCACTGGCAGCAGCCACTGGTAACAGGATTAGCAGAGCGAGGTATGTAGGCGGTGCTACAGAGTTCTTGAAGTGGTGGCCTAACTACGGCTACACTAGAAGAACAGTATTTGGTATCTGCGCTCTGCTGAAGCCAGTTACCTTCGGAAAAAGAGTTGGTAGCTCTTGATCCGGCAAACAAACCACCGCTGGTAGCGGTGGTTTTTTTGTTTGCAAGCAGCAGATTACGCGCAGAAAAAAAGGATCTCAAGAAGATCCTTTGATCTTTTCTACGGGGTCTGACGCTCAGTGGAACGAAAACTCACGTTAAGGGATTTTGGTCATGAGATTATCAAAAAGGATCTTCACCTAGATCCTTTTAAATTAAAAATGAAGTTTTAAATCAATCTAAAGTATATATGAGTAAACTTGGTCTGACAGTTACCAATGCTTAATCAGTGAGGCACCTATCTCAGCGATCTGTCTATTTCGTTCATCCATAGTTGCCTGACTCCCCGTCGTGTAGATAACTACGATACGGGAGGGCTTACCATCTGGCCCCAGTGCTGCAATGATACCGCGAGACCCACGCTCACCGGCTCCAGATTTATCAGCAATAAACCAGCCAGCCGGAAGGGCCGAGCGCAGAAGTGGTCCTGCAACTTTATCCGCCTCCATCCAGTCTATTAATTGTTGCCGGGAAGCTAGAGTAAGTAGTTCGCCAGTTAATAGTTTGCGCAACGTTGTTGCCATTGCTACAGGCATCGTGGTGTCACGCTCGTCGTTTGGTATGGCTTCATTCAGCTCCGGTTCCCAACGATCAAGGCGAGTTACATGATCCCCCATGTTGTGCAAAAAAGCGGTTAGCTCCTTCGGTCCTCCGATCGTTGTCAGAAGTAAGTTGGCCGCAGTGTTATCACTCATGGTTATGGCAGCACTGCATAATTCTCTTACTGTCATGCCATCCGTAAGATGCTTTTCTGTGACTGGTGAGTACTCAACCAAGTCATTCTGAGAATAGTGTATGCGGCGACCGAGTTGCTCTTGCCCGGCGTCAATACGGGATAATACCGCGCCACATAGCAGAACTTTAAAAGTGCTCATCATTGGAAAACGTTCTTCGGGGCGAAAACTCTCAAGGATCTTACCGCTGTTGAGATCCAGTTCGATGTAACCCACTCGTGCACCCAACTGATCTTCAGCATCTTTTACTTTCACCAGCGTTTCTGGGTGAGCAAAAACAGGAAGGCAAAATGCCGCAAAAAAGGGAATAAGGGCGACACGGAAATGTTGAATACTCATACTCTTCCTTTTTCAATATTATTGAAGCATTTATCAGGGTTATTGTCTCATGAGCGGATACATATTTGAATGTATTTAGAAAAATAAACAAATAGGGGTTCCGCGCACATTTCCCCGAAAAGTGCCACCTGATGCGGTGTGAAATACCGCACAGATGCGTAAGGAGAAAATACCGCATCAGGAAATTGTAAGCGTTAATATTTTGTTAAAATTCGCGTTAAATTTTTGTTAAATCAGCTCATTTTTTAACCAATAGGCCGAAATCGGCAAAATCCCTTATAAATCAAAAGAATAGACCGAGATAGGGTTGAGTGTTGTTCCAGTTTGGAACAAGAGTCCACTATTAAAGAACGTGGACTCCAACGTCAAAGGGCGAAAAACCGTCTATCAGGGCGATGGCCCACTACGTGAACCATCACCCTAATCAAGTTTTTTGGGGTCGAGGTGCCGTAAAGCACTAAATCGGAACCCTAAAGGGAGCCCCCGATTTAGAGCTTGACGGGGAAAGCCGGCGAACGTGGCGAGAAAGGAAGGGAAGAAAGCGAAAGGAGCGGGCGCTAGGGCGCTGGCAAGTGTAGCGGTCACGCTGCGCGTAACCACCACACCCGCCGCGCTTAATGCGCCGCTACAGGGCGCGTCCATTCGCCATTCAGGCTGCGCAACTGTTGGGAAGGGCGATCGGTGCGGGCCTCTTCGCTATTACGCCAGCTGGCGAAAGGGGGATGTGCTGCAAGGCGATTAAGTTGGGTAACGCCAGGGTTTTCCCAGTCACGACGTTGTAAAACGACGGCCAGTGAATTGTAATACGACTCACTATAGGGCGAATTGGGCCCAGAGGTGTAAAGTACTTGAGTAATTTTACTTGATTACTGTACTTAAGTATTATTTTTGGGGATTTTTACTTTACTTGAGTACAATTAAAAATCAATACTTTTACTTTTACTTAATTACATTTTTTTAGAAAAAAAAGTACTTTTTACTCCTTACAATTTTATTTACAGTCAAAAAGTACTTATTTTTTGGAGATCACTTCATTCTATTTTCCCTTGCTATTACCAAACCAATTGAATTGCGCTGATGCCCAGTTTAATTTAAATAGATCTGGCCATCTAGAGCGGCCGCGCGCACTAGTGAATTCCATGGATCGCGTTAAGATACATTGATGAGTTTGGACAAACCACAACTAGAATGCAGTGAAAAAAATGCTTTATTTGTGAAATTTGTGATGCTATTGCTTTATTTGTAACCATTATAAGCTGCAATAAACAAGTTAACAACAACAATTGCATTCATTTTATGTTTCAGGTTCAGGGGGAGGTGTGGGAGGTTTTTTAAAGCAAGTAAAACCTCTACAAATGTGGTATGGCTGATTATGATCAGTTATCTAGATCCGGTGGATCCTCAACCAGGATTTTCTTCAACATCACCAGCTTGCTTAAGCAAGGAGAAATTAGTAGCTCCGGATCCCTTGTACAGCTCGTCCATGCCGAGAGTGATCCCGGCGGCGGTCACGAACTCCAGCAGGACCATGTGATCGCGCTTCTCGTTGGGGTCTTTGCTCAGGGCGGACTGGGTGCTCAGGTAGTGGTTGTCGGGCAGCAGCACGGGGCCGTCGCCGATGGGGGTGTTCTGCTGGTAGTGGTCGGCGAGCTGCACGCTGCCGTCCTCGATGTTGTGGCGGATCTTGAAGTTCACCTTGATGCCGTTCTTCTGCTTGTCGGCCATGATATAGACGTTGTGGCTGTTGTAGTTGTACTCCAGCTTGTGCCCCAGGATGTTGCCGTCCTCCTTGAAGTCGATGCCCTTCAGCTCGATGCGGTTCACCAGGGTGTCGCCCTCGAACTTCACCTCGGCGCGGGTCTTGTAGTTGCCGTCGTCCTTGAAGAAGATGGTGCGCTCCTGGACGTAGCCTTCGGGCATGGCGGACTTGAAGAAGTCGTGCTGCTTCATGTGGTCGGGGTAGCGGCTGAAGCACTGCACGCCGTAGGTCAGGGTGGTCACGAGGGTGGGCCAGGGCACGGGCAGCTTGCCGGTGGTGCAGATGAACTTCAGGGTCAGCTTGCCGTAGGTGGCATCGCCCTCGCCCTCGCCGGACACGCTGAACTTGTGGCCGTTTACGTCGCCGTCCAGCTCGACCAGGATGGGCACCACCCCGGTGAACAGCTCCTCGCCCTTGCTCACCATGGTGGCACCGGTCAAAAAAAGAGACATACTGCATGTGGCAAAGTCCATAACCACTTTTCCTTCCAGTATAAAGGCCCACATTGCATCCATAATGAGGTTCTTTAGACATCTTCACACACGACAGTCAGCAGGCCAACATCTTCTGCACTGAAGTCCGACTCTACAATTACATCAAAACACATCCTGAAAATTTCTTTAGACTCATCCCTCTAAAGGACTGAAAGACCCCGGAAAACTGCTGCCAGATGACCTTTGAATTAACATGTTGAAAATTTCTTCTAGTTTTCTTCTGCAATTTTCTCCTGTTTTTTTTTTTTTTTATTTCAATCAAACGAGCAGTGTGTGTCCAATGCAGCGTAGAACTTGGATCTCACATTTCTTCCTGATATTCGATTGAATTCTTCTGCAGCACTTGCATCAGCTGGTGTTTCCTCTATGACTTCCTTCTGGATGTCAATTTCCACTGCATATCAGCTCACAAACTGGTAGTCTTGATTTACAGCAGTACAT

**3 Supplementary Figures.**

**3.1 Death rate and dose-response morphology of EAE zebrafish larvae.**

**Figure S1. Effects of EAE on zebrafish systemic development.** (A) Death rate of 250 µM EAE across exposure durations. n = 3 clutches (50 embryos each). (B) Representative bright-field images of EAE zebrafish larvae. Scale bar, 1000 µm. Data are mean ± S.E.M. ^*^*P<*0.05, ^**^*P*<0.01 *vs*. control, one-way analysis of variance (ANOVA), followed by Tukey’ s multiple comparison test.

**3.2 Effects of low-dose EAE on zebrafish larvae.**

**Figure S2. Effects of low-dose EAE on 120 hpf zebrafish larvae gene expression.** (A) Relative gene expression of liver, interrenal, brain and heart. Data are mean ± S.E.M. ^*^*P<*0.05, ^**^*P*<0.01 *vs*. control, Unpaired *t*-test for two independent group.

**3.3 Metabolomic and transcriptomic analysis of zebrafish larvae.**

**Figure S3. Metabolomic and transcriptomic analysis of 120 hpf 250 μM EAE zebrafish larvae.** (A) Heat map of differential metabolites. (B) KEGG enrichment (metabolomics). (C) Differential-metabolite list. (D) Volcano plot (transcriptomics). (E) KEGG enrichment (transcriptomics). (F) Spearman correlation chord diagram of differential genes and differential metabolites. (G) Correlation CirclePlot for differential genes and differential metabolites. (H) Spearman correlation network interaction diagram of differential genes and differential metabolites. (I) Interaction map of CCAcorrelation network between differential genes and differential metabolites. (J) Overlap of KEGG pathways between metabolomics and transcriptomics. (K) Heat map of differentially expressed lncRNAs. (L) Relative PCALRx expression. (M-O) Pearson correlations between PCALRx and L-aspartate, DL-malic acid and Citric. Correlation coefficients and two-tailed P values are shown. n = 6.

**3.4 Correlation analysis between significantly altered genes and organ-specific functional markers.**

**Figure S4. Correlation analysis between significantly altered genes and organ-specific functional markers.** (A1–G5) Pearson correlations between candidate genes (*kcnj1a.4, gck, PCALRx, slc26a1, fam21a, ptprna, sst2*) and organ functional markers using transcriptomic TPM values (n = 6). Correlation coefficients and two-tailed *P* values are shown.

**3.5 Effects of lncRNA PCALRx overexpression and knockdown.**

**Figure S5. Effects of lncRNA PCALRx overexpression and knockdown on 120 hpf zebrafish.** **PCALRx overexpression:** (A) PCALRx RNA-FISH staining after over-expression. Scale bar, 300 μm. (B) Death rate (0–120 hpf) across overexpression doses (n = 3 clutches; 50 embryos each). (C) Relative organ marker expression of control validation. (D, E) Body and head length after overexpression. (F–R) Relative organ marker expression after overexpression (liver: *gata6, foxa3, hhex, hmgcr, fasn*; interrenal: *nr5a1a, cyp17a1, star*; heart: *gata4, amhc, vmhc*; brain: *sox2*, *syn1*). (S,T) Behavioral radar plots. **PCALRx knockdown:** (U) Body length. (V–h) Relative organ marker expression. (i) Representative tracks. Data are mean ± S.E.M. n = 6 unless indicated. ^*^*P<*0.05, ^**^*P*<0.01 *vs*. control (unpaired *t*-test).

**3.6 Interaction between lncRNA PCALRx and pyruvate carboxylase.**

**Figure S6.** **Interaction between lncRNA PCALRx and pyruvate carboxylase**. (A) KEGG classification of identified proteins by LC-MS/MS. (B) Quantitative analysis of PCALRx FISH fluorescence and PC immunofluorescence optical density in amoxicillin treated ZF4 cell line, n=6. (B) Global protein ubiquitination levels of ZF4 cells. Data are mean ± S.E.M. Unpaired *t*-test for two independent groups, and one-way analysis of variance (ANOVA), followed by Tukey’ s multiple comparison test for more than two independent samples.

**3.7 Effects of drug repurposing candidate compounds on ZF4 cell line and zebrafish larvae.**

**Figure S7.** **Effects of drug repurposing candidate compounds on ZF4 cell line and zebrafish larvae**. (A–H) Dose–response effects of candidate compounds on oxaloacetate in ZF4 cells (n = 4). (I–X) Body and head length of 3–5 dpf larvae co-exposed to amoxicillin (250 µM) and each compound (100 µM). (Y) Representative larvae images. Data are mean ± S.E.M. n = 3 unless indicated. Unpaired *t*-test for two independent groups, and one-way analysis of variance (ANOVA), followed by Tukey’ s multiple comparison test for more than two independent samples.

**3.8 Pregnancy outcomes and organ markers in prenatal amoxicillin exposure offspring of mice.**

**Figure S8.** **Pregnancy outcomes and organ markers in prenatal amoxicillin exposure offspring of mice**. (A–D) GD20 fetal body weight, liver/body-weight ratio, body length, and tail length. (E) The homology comparison score of the entire sequence of PCALRx in the lncRNA database. (F–R) Organ marker expression in GD20 tissues (liver/heart/adrenal/brain panels as indicated). Data are mean ± S.E.M. n = 6 unless indicated. Unpaired *t*-test for two independent groups, and one-way analysis of variance (ANOVA), followed by Tukey’ s multiple comparison test for more than two independent samples. C, control; Am, prenatal amoxicillin exposure; VbL/VbH, Am + low/high vitamin B1. GD, gestational day.

**3.9 RNA-FISH staining of mouse organ and effect of PC knockdown in zebrafish larvae.**

**Figure S9.** **RNA-FISH staining of mouse organ and effect of PC knockdown in zebrafish larvae.** (A,B) RNA-FISH staining in GD20 fetal mice liver and heart. Scale bar, 50 µm. (C) H&E, PCALRx FISH, and PC (green) / TOMM20 (red) immunofluorescence in GD20 mice liver. Scale bar, 100 µm. The homology comparison score of the entire sequence of PCALRx in the lncRNA database. (D) RNA-FISH staining in GD20 fetal mice liver and adrenal. Scale bar, 100 µm. (E) Quantitative analysis of PCALRx FISH fluorescence optical density in amoxicillin and vitamin B1 treated mice liver, n=6. (F) Quantitative analysis of PC and TOMM20 immunofluorescence optical density in amoxicillin and vitamin B1 treated mice liver, n=6. (G) Quantitative analysis of PCALRx FISH fluorescence optical density in amoxicillin and vitamin B1 treated mice heart, n=6. (H) Quantitative analysis of PC and TOMM20 immunofluorescence optical density in amoxicillin and vitamin B1 treated mice heart, n=6. (I) Quantitative analysis of PCALRx FISH fluorescence optical density in amoxicillin and vitamin B1 treated mice adrenal, n=6. (J) Quantitative analysis of PC and TOMM20 immunofluorescence optical density in amoxicillin and vitamin B1 treated mice adrenal, n=6. (K) 120 hpf zebrafish larvae PC knockdown validation western blot. (L) Heat map of organ markers in 120 hpf zebrafish larvae under EAE, VB1 supplementary and PC knockdown. Data are mean ± S.E.M. One-way analysis of variance (ANOVA), followed by Tukey’ s multiple comparison test for more than two independent samples. C, control; Am, prenatal amoxicillin exposure; VbL/VbH, Am + low/high vitamin B1. GD, gestational day. KD, knockdown.
